# Lipid Metabolism and its Mechanism Triggered by Supercritical CO_2_ Extract of Adlay (*Coix lacryma-jobi var. ma-yuen* (Rom. Caill.) Stapf) Bran in High-Fat Diet Induced Hyperlipidemic Hamsters

**DOI:** 10.3389/fphar.2021.785944

**Published:** 2021-11-17

**Authors:** Chiao-Chih Huang, Tzu-Ching Lin, Chiung-Hui Liu, Hao-Chun Hu, Szu-Yin Yu, Shu-Jing Wu, Ming-Hong Yen, Yi-Hong Tsai, Fang-Rong Chang

**Affiliations:** ^1^ Graduate Institute of Natural Products, College of Pharmacy, Kaohsiung Medical University, Kaohsiung, Taiwan; ^2^ Department of Nutritional Health, Chia-Nan University of Pharmacy and Science, Tainan, Taiwan; ^3^ Department of Pharmacy and Master Program, Collage of Pharmacy and Health Care, Tajen University, Pingtung County, Taiwan; ^4^ Drug Development and Value Creation Research Center, Kaohsiung Medical University, Kaohsiung, Taiwan; ^5^ Department of Medical Research, Kaohsiung Medical University Hospital, Kaohsiung Medical University, Kaohsiung, Taiwan; ^6^ Department of Marine Biotechnology and Resources, National Sun Yat-sen University, Kaohsiung, Taiwan

**Keywords:** adlay bran, supercritical fluid extract, hypolipidemic capacity, unsaturated fatty acids (UFAs), ferulate phytostanol esters

## Abstract

Adlay (*Coix lacryma-jobi var. ma-yuen* (Rom. Caill.) Stapf) seeds are edible crop classified as Traditional Chinese Medicine (TCM). Adlay bran (AB) is one of the wastes generated during adlay refining processes. In this work, supercritical fluid extract of AB (AB-SCF) was investigated to reveal its lipid regulating potential and decode its bifunctional ingredients. AB-SCF×0.5 (30.84 mg/kg/body weight), AB-SCF×1 (61.67 mg/kg/BW), AB-SCF×5 (308.35 mg/kg/BW) and AB-SCF×10 (616.70 mg/kg/BW) were administrated to high fat-diet (HFD) induced hyperglycemic hamsters for 8 weeks. The results indicates that AB-SCF displays a prevention of dramatic body weight gains, lower levels of serum TG, TC, LDL-C and higher in HDL-C, amelioration of cardiovascular risk, alleviation of hepatic TG, TC and lipid peroxidation, and enhancement on cholesterol metabolism with higher bile acid excretion. Investigations on energy metabolic mechanism demonstrates that the hyperlipidemia mitigating capacities of AB-SCF are up-regulated on lipoprotein lipase, AMPK, p-AMPK and down-regulated at fatty acid synthase. Major bio-functional lipid compositions are identified as linoleic acid (28.59%) and oleic acid (56.95%). Non-lipid chemical and active markers are confirmed as 3-*O*-(*trans*-4-feruloyl)-*β*-sitostanol (1463.42 ppm), 3-*O*-(*cis*-4-feruloyl)-*β*-sitostanol (162.60 ppm), and *β*-sitosterol (4117.72 ppm). These compositions might synergistically responsible for the mentioned activities and can be regarded as analytical targets in quality control. AB-SCF may be considered as a promising complementary supplement, and developed as a functional food or new botanical drug in the future.

## Introduction

Adlay (*Coix lacryma-jobi var. ma-yuen* (Rom. Caill.) Stapf), an annual crop, distributed and had been widely cultivated around Asian centuries. Adlay seeds, the dehulled and polished endosperm, have long been used as edible crop with both medicinal and nutritious properties for thousands of decades, even classified as a Traditional Chinese Medicine, TCM. It was applied to relieve edema, warts, chapped skin, beriberi, neuralgia, edema, dysuria, hypertension, rheumatism, damp arthralgia and contracture of tendons and vessels, diarrhea due to spleen deficiency (Kuo et al., 2012; Wang et al., 2016; Li et al., 2017; Xu et al., 2017). Adlay seeds has also been processed as gluten-free products which were popularly available nowadays in the nutritious supplement market (Comino et al., 2013).

However, the procedures of manufacturing “polished adlay” are relatively a high-cost matter with causing large amounts of wastes. Since the concept of circular bioeconomy (CBE) is increasingly noticed and becomes a prominence trend (Stegmann et al., 2020). Adlay bran (AB) is one of those byproducts of “polished adlay” refining processes worthy to be salvaged and developed. In the recent decade, more and more researches had indicated that AB might be an important resource apart from the polished adlay used traditionally. It may be developed as health-promoting products, along with additional benefits to reduce production waste and increase the economic value during adlay processing (Chang et al., 2020). For example, lactams, spiroenones, ferulic acid and flavonoids from EtOAc soluble fractions of AB alcoholic extracts showed *in vitro* and *in vivo* capacities against the formation and proliferation of breast, lung and colon cancers. The probable mechanism might work through the delay of carcinogenesis by suppressing chronic inflammation (Lee et al., 2008; Chung et al., 2010; Chung et al., 2011a; Chen et al., 2011; Li et al., 2011; Huang et al., 2014). Caffeic and chlorogenic acids were the other major compounds identified in the same fraction with the suppressive effects on the growth of human gastric adenocarcinoma cell-line (AGS) and ulcer index (UI) (Chung et al., 2011b). Furthermore, luteolin as well as phenolic acids of the fraction were proven to exert allergic immune-regulatory effects and can probably be used to treat rheumatism (Chen et al., 2012a; Chen et al., 2012b). Sinapic acid, a special phenolic acid identified in AB methanol extract, majorly possessed the strong xanthine oxidase inhibitory activity to prevent the incidence of hyperuricemia (Zhao et al., 2014; Lin et al., 2018). In dermatological utilizations, the pressed AB oil was reported to be used against hyperpigmentation through the reductions of tyrosinase activity and melanin synthesis (Ting et al., 2019). Moreover, a clinical investigation demonstrated that orally administrated AB ethanol extract may prevent breast cancer patients suffered from severe acute radiation dermatitis after radiotherapy (Huang et al., 2015).

In a small scale of screen aiming at discovering new entities with bioactive potential, we prelimilarily found that supercritical fluid extracted aldlay bran (AB-SCF) showed blood-lipid regulating effect in hyperlipidemic hamsters (*n* = 3, data not shown). The use of supercritical carbon dioxide (SC-CO_2_) is an attractive alternative for organic solvents as “green” chemistry and classified as GRAS (Generally Recognized as Safe) by the Food and Drug Administration of the United States (US-FDA, 2008; Ramsey et al., 2009; Hsieh et al., 2012).

According to the literature survey, there was still no report focused on AB-SCF in revealing its anti-dyslipidemic/hypercholesterolemic capacities and the bioactive-responsible ingredients. The specific aims of this work were included as: *1*) to evaluate the serum and hepatic lipid regulating potential of AB-SCF on high-fat diet (HFD) induced hyperlipidemic hamsters, *2*) to clarify the lipid and energy metabolic mechanism triggered by AB-SCF, and *3*) to identify the nutritional and chemical compositions of AB-SCF.

In order to clarify the relationship between *in vivo* blood-lipid regulating effects and major compounds of AB-SCF, we hereby conducted evidence-guided column chromatography to isolate and identify its substances. Lipid compositions and its analytical fingerprint were further established by gas chromatography (GC) system. A comprehensive study, including bio-functional evidences and analytical properties of AB-SCF, were carried out in this work.

## Materials and Methods

### Plant Materials and Reagents

**FIGURE 1:**
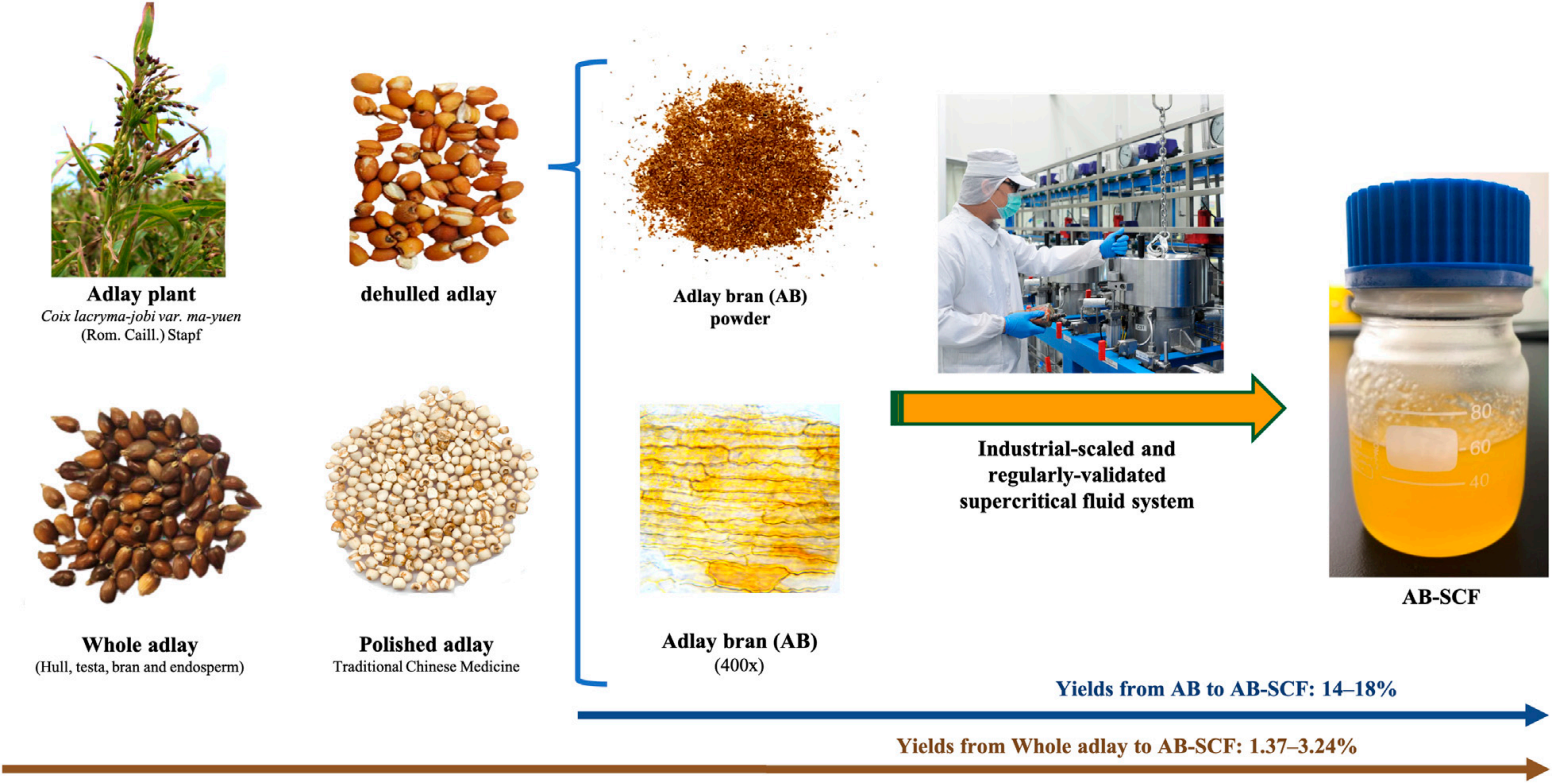
Adlay, adlay bran and the AB-SCF manufacturing processes. The yields of AB-SCF from AB were averagely 14–18%. The recovery of AB-SCF from whole adlay seeds was 1.37–3.24%. AB-SCF was extracted by an industrial-scaled and regularly-validated supercritical fluid system (NATEX Process Technology GmbH, Ternitz, Austria) settled in the Joben Bio-Medical Co., Ltd. (Pingtung, Taiwan).

Adlay seeds (*Coix lacryma-jobi var. ma-yuen* (Rom. Caill.) Stapf) were purchased in 2016 from the Daya District Farmers’ Association, which is directed by Taichung District Agricultural Research and Extension Station, Council of Agriculture, Taiwan. The raw plant material (batch number: 3A0015) was identified by Dr. Ming-Hong Yen. A voucher specimen (code no. KMU-Coix 001) was stored in the Graduate Institute of Natural Products, College of Pharmacy, Kaohsiung Medical University and Joben Bio-Medical Co., Ltd., Taiwan. The exact name of plant material has been checked on the authoritative website in Taxonomy: http://www.worldfloraonline.org. *Coix lacryma-jobi var. ma-yuen* (Rom. Caill.) Stapf was indicated as an accepted name in genus *Coix* (family Poaceae). The seeds were dried at room temperature by an air circulator and dehulled with a grinding mill. The grinded particles of adlay were then separated with an industrial-designed AB collector to obtain the AB. It was further grinded into powder and sieved through 20-mesh (aperture = 0.84 mm) for supercritical fluid extraction. The recovery of AB from whole adlay seeds (including hull, testa, bran and endosperm) was 9.8–10.8% (Figure 1).

Hepatic TG and TC were measured by ELISA kits, No. 10010303 and No. 10007640 purchased from Cayman Chemical Company (Ann Arbor, MI, United States), respectively. Fecal bile acid was analyzed with the Bile Acids kit (Product No. 450) obtained from Trinity Biotech Plc. (Wicklow, Leinster, Ireland). The anti-AMPK, anti-p-AMPK, anti-rabbit IgG, and mouse IgG bodies were purchased from Cell Signaling Technology Inc. (Danvers, MA, United States). Anti-FAS, anti-LPL, and anti-β-actin bodies were obtained from Abcam Inc. (Cambridge, MA, United States). Standard laboratory chow diet (No. 5001) was purchased from PMI^®^ Nutrition International (Brentwood, MO, United States). Standard mixtures for qualitative and quantitative analysis, F.A.M.E (Fatty acid methyl esters) Mix RM-4 (methyl linoleate, methyl oleate, methyl palmitate, methyl stearate), were purchased from Supelco^®^ (Sigma-Aldrich, St. Louis, MO, United States). Experimental animals in this work were fed either the standard chow diet or the HFD adapted from previous study (Yu et al., 2011). The nutrition facts of the standard chow diet were 3.36 kcal/g, containing 58.0% carbohydrates, 28.5% proteins and 13.5% fats. The HFD was 3.93 kcal/g, containing 44.53% carbohydrates, 21.88% proteins and 33.59% fats in one portion of 89.8% (wt/wt). 10% (wt/wt) of lard and 0.2% (wt/wt) of cholesterol were the rest of ingredients added, respectively (Sigma-Aldrich, St. Louis, MO, United States).

### Supercritical Fluid Extraction of AB-SCF

AB-SCF was extracted by an industrial-scaled and regularly-validated supercritical fluid system (NATEX Process Technology GmbH, Ternitz, Austria) settled in the Joben Bio-Medical Co., Ltd. (Pingtung, Taiwan). 2–3 kg AB for each batch was weighed accurately and supplied into the instrumental vessel to process the extraction. Detail parameters for the SCF solvent, SC-CO_2_, were optimized as 30–35 MPa and 40–60°C at a flow rate of 30–35 kg CO_2_ per hour, along with 60–75 min period for the balance between the recovery and production capacity. Every extraction was terminated depending on whether the yield was less than 0.1%. The yields of AB-SCF from AB were averagely 14–18%. The recovery of AB-SCF from whole adlay seeds was 1.37–3.24% (Figure 1).

### Animals and the *In Vivo* Experimental Design

Lipid metabolism of hamsters have been reported that closely resemble to human beings. Thus, hamsters were usually considered as the first and appropriate animal model for estimating hypolipidemic effects (Suica et al., 2016). Male Golden Syrian hamsters (6 weeks old) were purchased from the National Laboratory Animal Center (NLAC), Taipei City, Taiwan. All of them were housed under standard temperature (25 ± 1°C) and 50–60% relative humidity of conditions with a 12 h/12 h light-dark cycle. Standard chow diet and distilled water were provided *ad libitum*. Before the initiation of experiments, the hamsters were accommodated for 1 week to be stabilized and familiarize to the environment. All animal experimental protocols were supervised by the institutional animal care and use committee (IACUC) of Chia-Nan university (Tainan, Taiwan). The study conformed to the guidelines of the protocol CN-IACUC-105008R approved by the IACUC ethics committee.

The human equivalent dose (HED) of AB-SCF for hamsters were converted from the recommended daily dose for an adult human (assumed as 60 kg) which is 500 mg per day (one capsule/serving/day). HED between human and hamsters were calculated with a conversion coefficient, i.e., 7.4, based on body surface area (issued by the US Food and Drug Administration: http://www.fda.gov/downloads/Drugs/GuidanceComplianceRegulatoryInformation/Guidances/ucm078932.pdf). The formula would be as follows:

HED of hamsters = (Recommended daily dose of human/kg) × 7.4 (coefficient) = [500 (mg)/60 (kg)] × 7.4 = 61.67 mg/kg (designed as 1×)

After 1-week acclimatization, 70 hamsters were randomly divided into seven groups (*n* = 10/each group): C, a blank control group fed with standard chow diet and water; HFD, an HFD induced hyperlipidemic group fed with water; EM, a reference group for the vehicle (solubilizer) of AB-SCF fed with HFD and emulsifier (prepared with Tween 80/Span 80 in the ratio of 5:1 v/v, then diluted with sterilized RO water to 5%). Experimental groups were induced with HFD and administrated AB-SCF/emulsifier with a dose-ascending manner, i.e., AB-SCF×0.5, 30.84 mg/kg/BW (body weight); AB-SCF×1, 61.67 mg/kg/BW; AB-SCF×5, 308.35 mg/kg/BW; AB-SCF×10, 616.70 mg/kg/BW. The food intakes and water consumptions were daily monitored. The body weights were recorded weekly. All hamsters were fast for 16 h and sacrificed with 95% CO_2_ asphyxiation after the complete experimental period of 8 weeks. Serum, hepatic and fecal biochemical data, along with the energy metabolic mechanism(s) from proteins of liver tissues were further investigated.

### Quantitation of Lipid and Lipoprotein Levels in Serum and Feces

Biochemical data related to serum lipid and lipoprotein levels, such as triglyceride (TG), total cholesterol (TC), low density lipoprotein cholesterol (LDL-C), high density lipoprotein cholesterol (HDL-C), LDL-C/HDL-C ratio (a predictor of cardiovascular risk, Jukema et al., 2005). Blood samples were collected with cardiac puncture and immediately centrifugated at 1500 × *g* (4°C) for 15 min in anticoagulant-treated tubes (Greiner Bio-One GmbH, Frickenhausen, Germany) to obtain the serum samples. Fecal samples were gathered within 2 days (48 h) before the termination. The feces were dried, powdered, weighted and extracted with Folch Solution (chloroform/methanol 2:1, vol/vol). An aliquot of the organic phase was then dried and resuspended in isopropyl alcohol (Mera et al., 2015). Lipid and lipoprotein profiles including TG, TC, HDL-C, LDL-C were measured by the use of an automated clinical chemistry analyzer, Fuji Dry-Chem 4000i, and its dedicated biochemical slides (Fujifilm, Tokyo, Japan).

### Measurement of Fecal Bile Acid

Fecal lipid and bile acid were assessed. The feces were dried, weight and then grounded as fine powder in a mechanical blender. Aliquots of ground feces were well-mixed with sodium borohydride and then subjected to strong alkaline hydrolysis at 120–130°C for 12 h. The extracted fecal bile acid was measured enzymatically measured with a commercial bile acids kit (Product No. 450-A from Trinity Biotech Plc., Wicklow, Leinster, Ireland).

### Analysis of Hepatic Triglyceride, Total Cholesterol Levels and the Oxidative Stress Markers

Hepatic lipid profiles (TC and TG) and oxidative stress markers i.e., MDA (malondialdehyde) and GSH (glutathione) were evaluated. All liver tissues were carefully collected, washed three times in ice cold saline, blotted individually on ash-free filter paper, weighted and aliquoted into few parts and frozen stored at −80°C. Before further analysis of hepatic TG and TC, each piece was homogenized with Tris-buffer (Sigma-Aldrich, St. Louis, MO, United States). The centrifuged supernatants were extracted by chloroform-isopropanol-NP40 (7:11:0.1, v/v) with a bullet blender (Lee et al., 2015). After the centrifugation again at 12,000 × *g* (4°C) for 10 min, hepatic TG and TC levels of the supernatants were measured in triplicate by using commercial enzymatic kits for TG (No. 10010303) and for TC (No. 10007640) from Cayman Chemical Company (Ann Arbor, MI, United States).

For the estimations of GSH and MDA, liver tissues were homogenized in phosphate buffer saline (PBS) 50 mM pH (7.4) and potassium phosphate buffer 10 mM pH (7.4), respectively. GSH levels were carried out with a commercial glutathione assay kit (product CS0260 from Sigma-Aldrich, St. Louis, MO, United States). This principle of MDA assessment depends on its formation as an end product of lipid peroxidation which reacts with thiobarbituric acid to produce thiobarbituric acid reactive substance (TBARS). TBARS, a pink chromogen, would be detected at 532 nm in a Spectrophotometry. A TEP (1,1,3,3-tetraethoxypropane) standard (Sigma-Aldrich, St. Louis, MO, United States) was used to build a standard curve against which readings of the samples were plotted (Noeman et al., 2011).

### Extraction of Liver Tissue Protein and Western Blot Analysis

Bio-markers related to energy-balance, lipoprotein metabolism and oxidative-stress in liver, e.g., AMPK (adenosine-monophosphate-activated protein kinase), p-AMPK (phosphorylated-AMPK), FAS (fatty acid synthase) and LPL (hepatic lipoprotein lipase) were investigated to elaborate the energy metabolic mechanism. Protein extraction was conducted by homogenizing each liver sample in 1 ml lysis buffer (containing 10 mM-HEPES, pH 7.8), 10 mM KCl, 2 mM MgCl_2_, 1 mM dithiothreitol (DTT), 0.1 mM EDTA, and 0.1 mM phenylmethylsulfonyl fluoride) at 4°C. Meanwhile, 80 μl of 10% NP-40 solution used for breaking the nuclear membrane within a cell was added as well. After the homogenization, the lysates were centrifuged for 2 min at 14,000 × *g*. Equal amounts of lysed protein (30 µg/lane) were loaded onto SDS-polyacrylamide gels, and electrophoretically transferred to a PVDF membrane (Bio-Rad Laboratories, Hercules, CA, United States). After blocking with 5% (w/v) skim milk in 0.1% (v/v) Tween 20-containing PBS (PBST) for 1 h at room temperature, the membrane was incubated with the following specific primary antibodies for 1 h at room temperature: anti-AMPK (1:1000), anti-phospho-AMPK (1:1000), anti-FAS (1:1000), anti-LPL (1:1000), and anti-β-actin (1:25000) antibodies (in 5% w/v skim milk with PBST). Antibody recognition was detected with the respective secondary antibody, either anti-mouse IgG or anti-rabbit IgG antibodies linked to horseradish peroxidase. Antibody-bound proteins were detected with the ECL western blotting analysis system (Amersham, Aylesbury, UK). The expression of β-actin was used as loading control. Relative protein expressions were quantified densitometrically with an AlphaImager 2200 (Alpha Innotech Corp., San Leandro, CA, United States), and processed using AlphaEaseFC software in referring to the β-actin reference bands in triplicate (Wei et al., 2017).

### Preparation of Fatty Acid Methyl Esters of AB-SCF for GC Analysis

An aqueous concentrated HCl (conc. HCl; 35%, w/w) catalyzation was conducted to prepare the fatty acid methyl esters (FAMEs) of AB-SCF for GC analysis (Ichihara and Fukubayashi, 2010). Briefly, an 8% (w/v) HCl prepared in methanol/water (85:15, v/v) was diluted in 9.7 ml of concentrated HCl with 41.5 ml of methanol. Toluene (0.2 ml), methanol (1.5 ml), and the 8% HCl solution (0.3 ml) were added sequentially to the AB-SCF. The final HCl concentration was 1.2% (w/v). This solution (2 ml) was incubated at 45°C overnight or heated at 100°C for 1.5 h. The catalyzed AB-SCF was dried, then dissolved in EtOAc, filtered through 0.22 µm filter and subjected to GC analysis.

### GC Analysis

The analysis of major compositions and the establishment of AB-SCF fingerprint were carried out with a gas chromatography system (Trace GC Ulture/ITQ 900, Thermo fisher Scientific, United States) with a flame ionization detector (FID). The capillary column was RT^®^-2560 (100 m × 250 μm  ×  0.2 μm) coated with biscyanopropyl polisiloxane as stationary phase (Restek Corporation, Bellefonte, PA, United States). The column oven temperature was programmed 150°C (held for 2 min), increased to 220°C at a rate of 35°C/min (held for 1 min), then raised to 225°C a rate of 0.5°C/min (maintained for 1 min). The other parameters were as follows: injection temperature, 225°C; detector temperature, 250°C; carrier gas, Helium at 1 ml/min; injection volume, 1 μl. The relative percentage of each major component in AB-SCF was quantified based on the peak area integrated by Thermo Xcalibur™ data analysis program (Thermo fisher Scientific, United States). Qualitative and quantitative analysis of AB-SCF (C16:0 Palmitate, C18:0 Stearate, C18:1 Oleate, C18:2 Linoleate) was carried out in comparing with the F.A.M.E Mix RM-4 standards.

### Separation, Isolation and Purification of Chemical Substances of AB-SCF

10.0614g (density = 0.922 g/ml) of AB-SCF was firstly subjected into a Sephadex^®^ LH-20 column in an environment of Dichloromethane (DCM):Methanol = 1:1 for gel filtration (Fine Chemicals AB, Uppsala, Pharmacia). Sephadex^®^ LH-20 is composed by cross-linked dextran for molecular sizing natural products in accordance to molecular weight. The purpose would be to divide the nonlipid substances from apolar/lipid mixtures (Wells and Dittmer, 1963). Seven fractions, AB-SCF-S1 to S8, were yielded after passing AB-SCF through the Sephadex^®^ LH-20 column. Thin-layer chromatography was monitored with silica gel 60 F_254_ and RP-18 F_254S_ TLC plates (Merck, Darmstadt, Germany) with substance visualized by 10% (v/v) H_2_SO_4_/ethanol spray (Supplementary Figure S1). After brief screen of ^1^H NMR (Nuclear Magnetic Resonance) spectroscopy on every AB-SCF-Sn fraction, AB-SCF-S1 was the main ingredient composed of 78.92% triglyceride, along with AB-SCF-S4 and S5 were detected 8.39% for mostly fatty acids. AB-SCF-S2 (192.50 mg, 1.91%) and S3 (282.27 mg, 2.86%) were observed with interesting minor signals different but still mixed with triglyceride and fatty acids. Meanwhile, the last three fractions, AB-SCF-S6 to S8, occupied only 0.58% (58.36 mg) in AB-SCF with signals of oil and complicate mixtures (Supplementary Figure S2).

AB-SCF-S3 was combined into AB-SCF-S2 as S2’ (totally 479.77 mg, 4.77% weight/total weight of eluents) and fractioned with silica gel (230–400 mesh, Merck, Darmstadt, Germany) open column and stepwise eluted with *n*-hexane:EtOAc from 80:1–100% into 17 subfractions (S2′-1–17). With the continuous monitor of ^1^H NMR, S2′-7 which was different from oil constituents were separated with another silica gel open column from the solvent system of *n*-hexane:EtOAc from 80:1–100%. S2′-7-1 to 5 were obtained. S2′-7-5-1 to 9 were further got carried out by silica preparative TLC *n*-Hexane:DCM (1:1). However, the amounts of these fractions were too low (all less than 2 mg) to be re-measured by ^1^H NMR. The other fraction considered not being fatty acids from S2′, the S2′-11, was directly processed by silica preparative TLC *n*-hexane: DCM (1:2) to yield two mixed geometric isomers, (**1)** (*R*f = 0.58) and (**2**) (*R*f = 0.58). (**3**) was filtered out from S2′-11-6 by using DCM and Methanol (*R*f = 0.38) (Supplementary Figures S3, S4).

### Statistical Analysis

Data were presented as mean ± standard deviation (SD) from different and independent experiments. Values were evaluated by one-way ANOVA, followed by Duncan’s multiple range test using the 9.0 Statistical Analysis System (SAS Institute, Cary, NC, United States). Difference was considered significant when *p*-value was <0.05.

## Results

### Effects of AB-SCF on Body Weight and Daily Food Intake in Hyperlipidemic Hamsters

The effects of AB-SCF×0.5, AB-SCF×1, AB-SCF×5, AB-SCF×10 on the changes of body weight (BW) and daily food intake (DFI) were recorded in the model of high-fat diet induced hyperlipidemic male hamsters (Table 1). After an 8-week administration, the external appearances and health conditions of all hamsters were remained ordinarily with no adverse effects observed. BWs were stable and steadily increased in each group. High fat diet induced groups were generally heavier than C with significance (*p* < 0.05). Meanwhile, AB-SCF×10 displayed significantly lower BW than EM, AB-SCF×5, AB-SCF×1 and AB-SCF×0.5 (*p* < 0.05), and close to C at the 6th to 8th week. The daily food intake (DFI) data showed no difference among all groups.

**TABLE 1:** Body weight (BW) gains and daily food intake (DFI) among normal and hyperlipidemic hamsters administrated with AB-SCF.

Body weight (BW)							
week	C (g)	HFD (g)	HFD (g)				
			EM	AB-SCF×0.5	AB-SCF×1	AB-SCF×5	AB-SCF×10
0	100.00 ± 4.88^ab^	104.40 ± 7.47^a^	97.50 ± 5.44^b^	99.30 ± 6.53^ab^	99.00 ± 4.81^ab^	98.90 ± 7.20^ab^	98.30 ± 2.06^ab^
1	115.80 ± 2.35^a^	110.90 ± 1.29^b^	108.00 ± 1.05^c^	105.20 ± 1.03^d^	101.70 ± 1.16^e^	100.40 ± 2.27^e^	101.00 ± 3.06^e^
2	119.40 ± 2.55^ab^	121.40 ± 4.97^a^	115.60 ± 9.91^bc^	116.10 ± 2.47^bc^	111.40 ± 2.41^cd^	107.90 ± 4.28^d^	108.50 ± 4.97^d^
3	122.30 ± 3.16^bc^	130.40 ± 8.37^a^	125.30 ± 8.62^ab^	122.90 ± 5.95^bc^	118.80 ± 2.49^c^	118.10 ± 6.26^c^	118.30 ± 5.74^c^
4	126.50 ± 4.17^b^	138.10 ± 9.05^a^	136.70 ± 9.43^a^	131.80 ± 6.65^ab^	127.30 ± 4.19^b^	129.60 ± 6.54^b^	127.00 ± 7.83^b^
5	130.90 ± 6.52^d^	147.60 ± 8.75^ab^	150.20 ± 9.27^a^	139.20 ± 7.21^bc^	139.20 ± 8.72^bc^	136.40 ± 7.49^cd^	133.00 ± 8.43^cd^
6	134.10 ± 5.70^c^	153.30 ± 10.09^a^	152.50 ± 7.59^a^	145.40 ± 8.40^ab^	142.80 ± 9.68^b^	143.20 ± 7.91^b^	135.20 ± 7.55^c^
7	138.50 ± 6.33^b^	159.50 ± 11.22^a^	164.10 ± 12.24^a^	156.20 ± 9.83^a^	146.30 ± 10.67^b^	145.40 ± 7.53^b^	142.50 ± 9.91^b^
8	141.10 ± 6.97^f^	164.60 ± 11.06^ab^	167.10 ± 11.37^a^	158.40 ± 8.46^bc^	152.90 ± 5.36^cd^	149.40 ± 10.59^de^	143.80 ± 7.21^ef^
**Daily food intake (DFI)**							
**week**	**C (g/day)**	**HFD (g/day)**	**HFD (g/day)**				
						**EM**	**AB-SCF×0.5**	**AB-SCF×1**	**AB-SCF×5**	**AB-SCF×10**
0	9.10 ± 0.57^a^	9.00 ± 0.67^a^	9.30 ± 1.25^a^	9.40 ± 0.97^a^	9.00 ± 0.47^a^	9.20 ± 0.79^a^	9.20 ± 1.14^a^
1	9.20 ± 1.32^a^	9.90 ± 2.64^a^	9.90 ± 1.85^a^	9.90 ± 1.29^a^	9.20 ± 1.03^a^	9.30 ± 1.57^a^	9.50 ± 0.85^a^
2	10.60 ± 2.17^a^	10.40 ± 1.26^a^	10.40 ± 2.59^a^	10.80 ± 1.93^a^	10.40 ± 2.17^a^	10.10 ± 1.10^a^	10.40 ± 1.84^a^
3	11.80 ± 1.87^a^	11.80 ± 1.99^a^	11.30 ± 1.64^a^	11.20 ± 1.32^a^	11.70 ± 1.34^a^	11.70 ± 0.95^a^	11.80 ± 1.40^a^
4	11.90 ± 1.29^a^	11.90 ± 1.37^a^	11.60 ± 1.65^a^	11.50 ± 1.78^a^	11.80 ± 2.25^a^	11.90 ± 1.20^a^	11.80 ± 1.62^a^
5	11.90 ± 1.20^a^	11.90 ± 1.20^a^	12.00 ± 1.49^a^	11.70 ± 1.64^a^	11.90 ± 1.52^a^	11.80 ± 0.92^a^	11.90 ± 1.37^a^
6	12.00 ± 0.82^a^	11.80 ± 0.92^a^	12.00 ± 0.67^a^	11.80 ± 1.03^a^	12.00 ± 0.82^a^	12.00 ± 1.05^a^	12.10 ± 1.10^a^
7	12.00 ± 0.67^a^	11.90 ± 0.74^a^	12.10 ± 1.66^a^	11.90 ± 0.99^a^	11.80 ± 1.87^a^	12.00 ± 0.82^a^	12.40 ± 1.35^a^
8	12.10 ± 1.66^a^	12.30 ± 2.31^a^	12.40 ± 3.27^a^	12.00 ± 2.98^a^	12.00 ± 2.31^a^	12.30 ± 2.00^a^	12.40 ± 1.07^a^

HFD, high-fat diet; EM, Tween 80/Span 80 = 5:1 v/v, diluted water to 5%; AB-SCF, supercritical fluid extracted aldlay bran. C, a blank control group fed with standard chow diet and water; HFD, a high fat diet induced hyperlipidemic group fed with water; EM, a high fat diet induced fed with the emulsifier of AB-SCF; Experimental groups were induced with HFD and administrated AB-SCF/emulsifier with a dose-ascending manner, i.e., AB-SCF×0.5, 30.84 mg/kg/BW; AB-SCF×1, 61.67 mg/kg/BW; AB-SCF×5, 308.35 mg/kg/BW; AB-SCF×10, 616.70 mg/kg/BW. Data are mean ± SD, *n* = 10 hamsters in each group. Means with different letters in the same column were significantly different at *p* < 0.05 as statistically analyzed by Duncan’s multiple range tests.

### Effects of AB-SCF on Lipid and Lipoprotein Levels in Serum and Feces of Hyperlipidemic Hamsters

After the 8 weeks administrations, effects of AB-SCF×0.5, AB-SCF×1, AB-SCF×5, and AB-SCF×10 groups on the changes of serum and fecal lipid and lipoprotein profiles were examined after the termination (Table 2). TG and TC levels of the C group were 47.30 ± 9.59 and 53.60 ± 3.44 mg/dl, respectively. However, HFD and EM had been increased significantly to 328.50 ± 30.15 and 360.90 ± 30.22 mg/dl (*p* < 0.05). All AB-SCF administrated groups, compared to the HFD and EM, exhibited markedly lower data in not only TG and TC, but also in LDL-C (*p* < 0.05). In addition, HDL-C levels were significantly higher (*p* < 0.05) in all AB-SCF groups than in HFD and EM. The ratios of LDL-C/HDL-C, a predictor of cardiovascular risk, were dose-dependently and meaningfully decreased (*p* < 0.05) in AB-SCF administrated hyperlipidemic hamsters as 2.60 ± 0.66, 1.69 ± 0.50, 0.49 ± 0.10, 0.48 ± 0.26, respectively (with 0.11 ± 0.04 in C, 4.73 ± 1.04 in HFD, and 4.30 ± 0.90 in EM).

**TABLE 2:** Serum and fecal lipid and lipoprotein profiles among normal and hyperlipidemic hamsters administrated with AB-SCF.

Serum										
	C			HFD	HFD				
					EM	AB-SCF×0.5	AB-SCF×1	AB-SCF×5	AB-SCF×10
TG (mg/dl)	47.30 ± 9.59^f^			328.50 ± 30.15^a^	286.20 ± 21.37^b^	216.60 ± 36.61^c^	153.90 ± 24.26^d^	142.90 ± 32.37^de^	135.40 ± 29.76^e^
TC (mg/dl)	53.60 ± 3.44^d^			360.90 ± 30.22^a^	372.90 ± 46.24^a^	236.30 ± 36.21^b^	214.50 ± 20.90^b^	148.30 ± 13.97^c^	143.20 ± 26.98^c^
HDL-C (mg/dl)	39.30 ± 2.54^d^			52.20 ± 4.73^c^	52.90 ± 4.28^c^	55.20 ± 7.45^c^	70.80 ± 8.74^b^	83.00 ± 9.24^a^	85.90 ± 9.06^a^
LDL-C (mg/dl)	4.10 ± 1.37^e^			242.90 ± 33.89^a^	225.60 ± 39.85^a^	140.70 ± 28.81^b^	116.20 ± 0.09^c^	40.60 ± 6.45^d^	40.20 ± 18.37^d^
LDL-C/HDL-C	0.11 ± 0.04^e^			4.73 ± 1.04^a^	4.30 ± 0.90^a^	2.60 ± 0.66^b^	1.69 ± 0.50^c^	0.49 ± 0.10^d^	0.48 ± 0.26^d^
**Feces**										
**Every 100 mg feces**		**C**	**HFD**		**HFD**				
										**EM**	**AB-SCF×0.5**	**AB-SCF×1**	**AB-SCF×5**	**AB-SCF×10**
TG (mg)		6.50 ± 1.29^c^	14.00 ± 1.41^b^		18.00 ± 1.25^a^	17.00 ± 1.4^a^	17.00 ± 1.41^a^	17.5 ± 2.12^a^	19.00 ± 1.41^a^
TC (mg)		11.00 ± 0.82^c^	35.00 ± 4.08^a^		34.00 ± 0.83^a^	39.00 ± 1.41^a^	27.00 ± 1.41^b^	26.5 ± 0.71^b^	26.00 ± 2.83^b^
LDL-C (mg)		0.18 ± 0.05^b^	0.35 ± 0.06^a^		0.33 ± 0.12^a^	0.35 ± 0.07^a^	0.30 ± 0.02^a^	0.35 ± 0.07^a^	0.35 ± 0.07^a^
HDL-C (mg)		0.55 ± 0.10^c^	1.25 ± 0.1^a^		1.05 ± 0.07^ab^	1.05 ± 0.06^ab^	1.05 ± 0.07^ab^	1.05 ± 0.05^ab^	1.05 ± 0.03^ab^
Bile Acids (μmol)		17.37 ± 1.46^e^	116.08 ± 9.73^c^		113.28 ± 3.10^c^	126.28 ± 7.10^cd^	167.42 ± 14.03^c^	236.91 ± 19.85^ab^	256.62 ± 11.45^a^

TG, triglyceride; TC, total cholesterol; LDL-C, low-density lipoprotein cholesterol; HDL-C, high-density lipoprotein cholesterol; HFD, high-fat diet; EM, Tween 80/Span 80 = 5:1 v/v, diluted water to 5%; AB-SCF, supercritical fluid extracted aldlay bran. C, a blank control group fed with standard chow diet and water; HFD, a high fat diet induced hyperlipidemic group fed with water; EM, a high fat diet induced fed with the emulsifier of AB-SCF; Experimental groups were induced with HFD and administrated AB-SCF/emulsifier with a dose-ascending manner, i.e., AB-SCF×0.5, 30.84 mg/kg/BW; AB-SCF×1, 61.67 mg/kg/BW; AB-SCF×5, 308.35 mg/kg/BW; AB-SCF×10, 616.70 mg/kg/BW. Data are mean ± SD, *n* = 10 hamsters in each group. Means with different letters in the same column were significantly different at *p* < 0.05 as statistically analyzed by Duncan’s multiple range tests.

In feces, the lipid and lipoprotein profiles were generally higher in high fat diet induced hyperlipidemic hamsters. Only TC were expressively alleviated (*p* < 0.05) by AB-SCF×1, AB-SCF×5, AB-SCF×10 with 27.00 ± 1.41 mg, 26.00 ± 2.83 mg, 26.5 ± 0.71 mg in every 100 mg feces (11.00 ± 0.82 from C, 35.00 ± 4.08 from HFD, 34.00 ± 0.83 from EM and 39.00 ± 1.41 from AB-SCF×0.5). Bile acids (μmol/100 mg feces) in HFD (116.08 ± 9.73) and EM (113.28 ± 3.10) was obviously elevated (*p* < 0.05) than in C (17.37 ± 1.46). AB-SCF groups would improve the excretions of bile acids with significances in dose-dependent manner.

### Effects of AB-SCF on Hepatic Triglyceride, Total Cholesterol Levels and the Oxidative Stress Markers of Hyperlipidemic Hamsters

**FIGURE 2:**
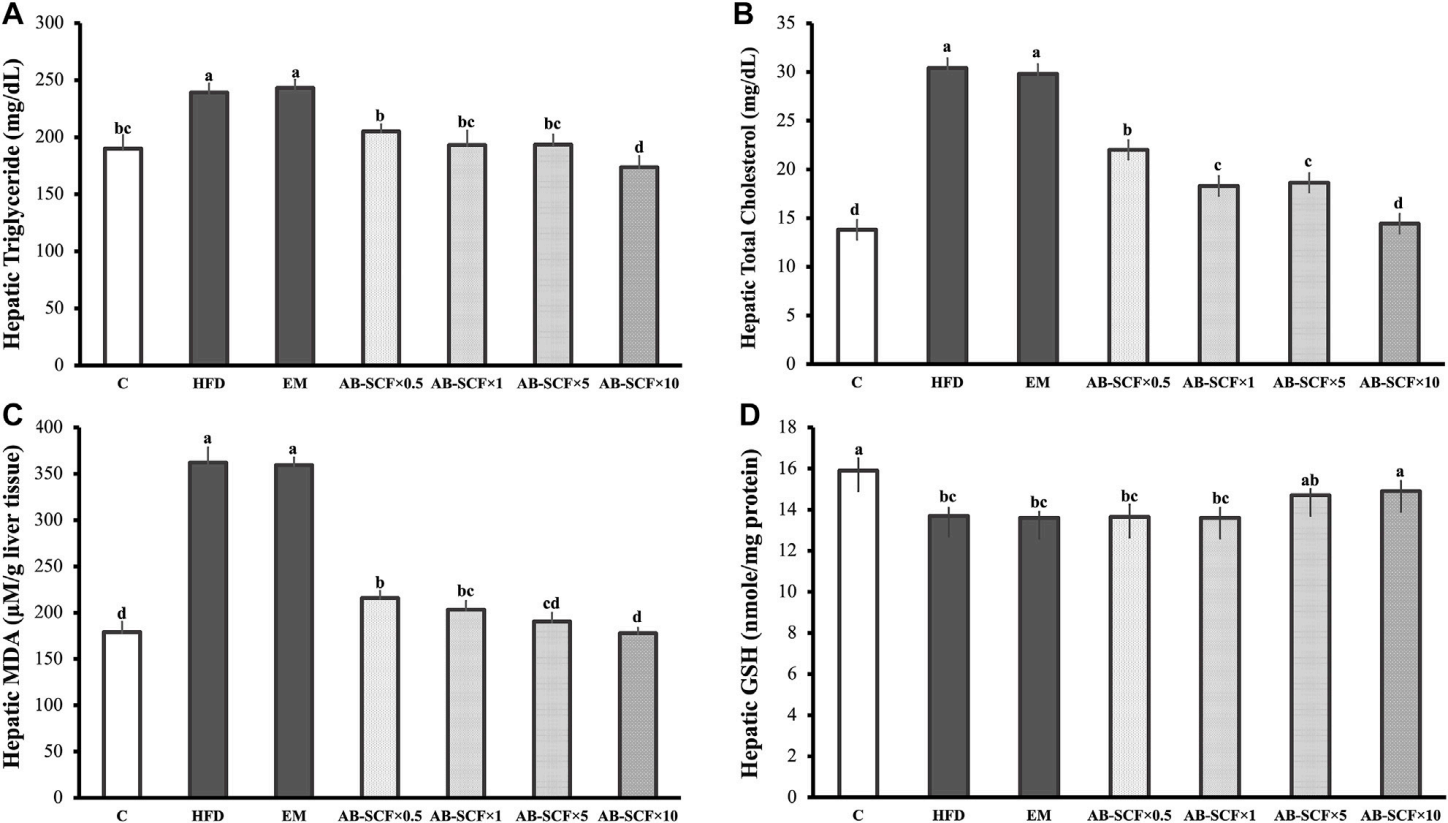
Effects of AB-SCF on the lipid accumulation and oxidative stresses in liver. **(A)** Levels of hepatic triglyceride; **(B)** Levels of hepatic total cholesterol; **(C)** Levels of hepatic MDA (malondialdehyde); **(D)** Levels of hepatic GSH (glutathione) in normal, HFD induced and AB-SCF administrated hyperlipidemia hamsters. HFD, high-fat diet; EM, Tween 80/Span 80 = 5:1 v/v, diluted water to 5%; AB-SCF, supercritical fluid extracted aldlay bran. C, a blank control group fed with standard chow diet and water; HFD, a high fat diet induced hyperlipidemic group fed with water; EM, a high fat diet induced fed with the emulsifier of AB-SCF; Experimental groups were induced with HFD and administrated AB-SCF/emulsifier with a dose-ascending manner, i.e., AB-SCF×0.5, 30.84 mg/kg/BW; AB-SCF×1, 61.67 mg/kg/BW; AB-SCF×5, 308.35 mg/kg/BW; AB-SCF×10, 616.70 mg/kg/BW. Data are mean ± SD, *n* = 10 hamsters in each group. Means with different letters in the same column were significantly different at *p* < 0.05 as statistically analyzed by Duncan’s multiple range tests.

Hepatic TG and TC were both aggravatedly higher in HFD and EM groups than the other ones (Table 3 and Figure 2). In contrast, AB-SCF had significantly ameliorated the levels of hepatic TG and TC (*p* < 0.05). Especially in the AB-SCF×10 group, hepatic TG and TC of hyperlipidemic hamsters were almost retrieved back to levels of the standard chow diet ones.

**TABLE 3:** Hepatic triglyceride, total cholesterol, GSH and MDA among hyperlipidemic hamsters administrated with AB-SCF.

	Hepatic TG (mg/dl)	Hepatic TC (mg/dl)	Hepatic MDA (μM/g liver tissue)	Hepatic GSH (nmole/mg protein)
C	189.90 ± 11.80^bc^	13.80 ± 1.91^d^	179.10 ± 11.30^d^	15.90 ± 0.60^a^
HFD	239.00 ± 8.01^a^	30.40 ± 2.47^a^	362.10 ± 16.4^a^	13.70 ± 0.40^bc^
EM	243.00 ± 7.30^a^	29.80 ± 1.23^a^	359.50 ± 8.30^a^	13.60 ± 0.30^bc^
AB-SCF×0.5	205.00 ± 6.20^b^	22.00 ± 1.03^b^	215.70 ± 7.80^b^	13.65 ± 0.60^bc^
AB-SCF×1	193.10 ± 12.39^bc^	18.30 ± 1.61^c^	203.20 ± 9.40^bc^	13.60 ± 0.50^bc^
AB-SCF×5	193.30 ± 8.93^bc^	18.63 ± 1.37^c^	190.50 ± 9.40^cd^	14.70 ± 0.30^ab^
AB-SCF×10	173.70 ± 9.65^d^	14.43 ± 1.16^d^	177.80 ± 5.80^d^	14.90 ± 0.50^a^

TG, triglyceride; TC, total cholesterol; MDA, malondialdehyde; GSH, glutathione; HFD, high-fat diet; EM, Tween 80/Span 80 = 5:1 v/v, diluted water to 5%; AB-SCF, supercritical fluid extracted aldlay bran. C, a blank control group fed with standard chow diet and water; HFD, a high fat diet induced hyperlipidemic group fed with water; EM, a high fat diet induced fed with the emulsifier of AB-SCF; Experimental groups were induced with HFD and administrated AB-SCF/emulsifier with a dose-ascending manner, i.e., AB-SCF×0.5, 30.84 mg/kg/BW; AB-SCF×1, 61.67 mg/kg/BW; AB-SCF×5, 308.35 mg/kg/BW; AB-SCF×10, 616.70 mg/kg/BW. Data are mean ± SD, *n* = 10 hamsters in each group. Means with different letters in the same column were significantly different at *p* < 0.05 as statistically analyzed by Duncan’s multiple range tests.

As compared to the C in the measurement of MDA and GSH (179.10 ± 11.30; 15.90 ± 0.60), HFD (362.10 ± 16.4; 13.70 ± 0.40) and EM (359.50 ± 8.30; 13.60 ± 0.30) showed significant increases and depletions, respectively. In lipid peroxidation of hyperlipidemic hamster livers, AB-SCF groups exhibited promising protective affections with dose-dependent manner (MDA: AB-SCF×0.5, 215.70 ± 7.80; AB-SCF×1, 203.20 ± 9.40; AB-SCF×5, 190.50 ± 9.40; AB-SCF×10, 177.80 ± 5.80. *p* < 0.05). Meanwhile, the GSH results were positively regulated in the relatively higher doses of AB-SCF×5 and AB-SCF×10 which were indicated as 14.70 ± 0.30 and 14.90 ± 0.50, respectively. Thus, AB-SCF might be able to be considered as an alleviating agent against the accumulation and lipid peroxidation in liver.

### Effects of AB-SCF Triggered Expressions of Hepatic Energy Metabolic Mechanism Through FAS, LPL, AMPK and p-AMPK Proteins in Hyperlipidemic Hamsters

**FIGURE 3:**
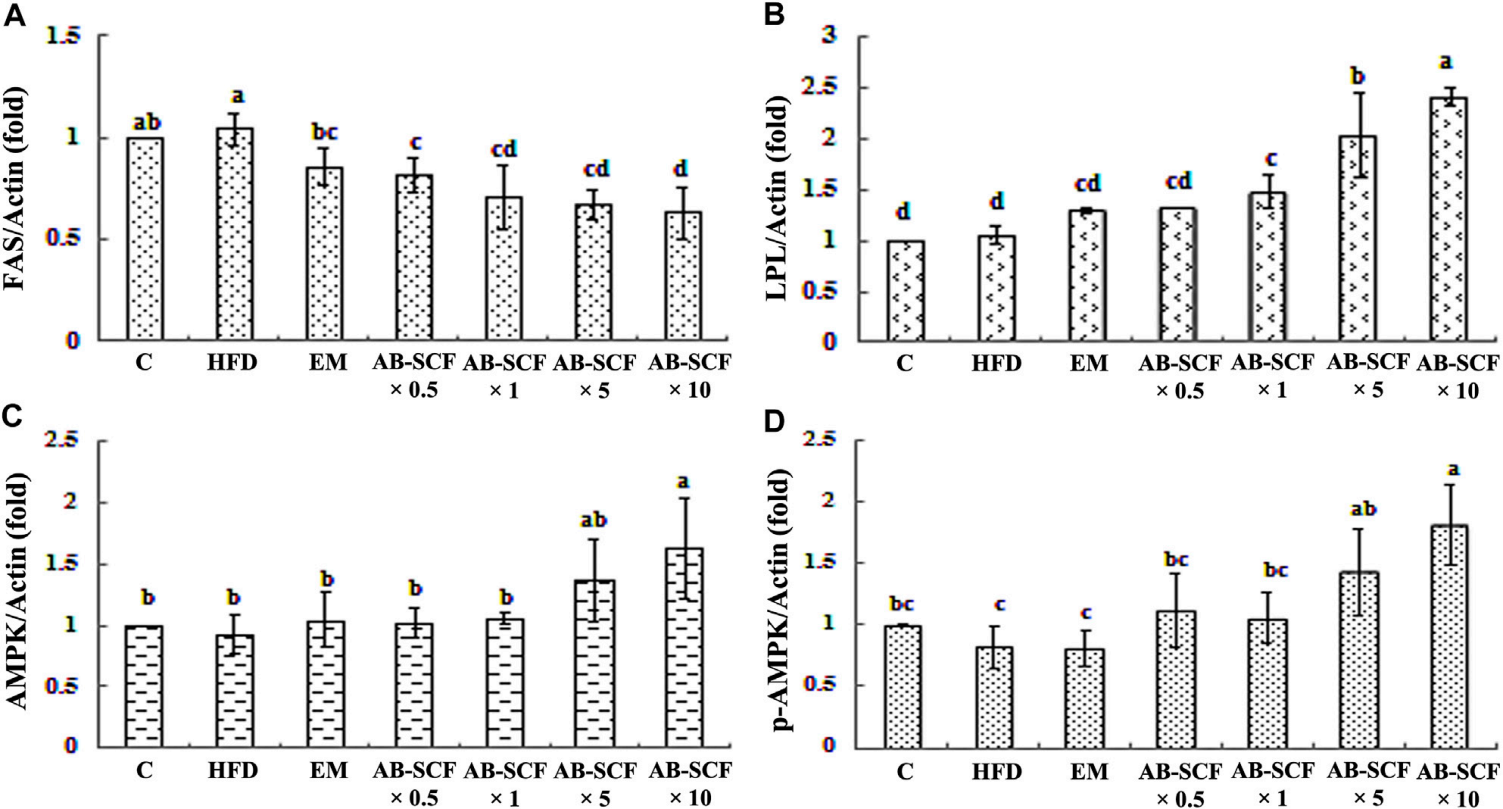
Effects of AB-SCF on the expression of FAS, LPL, AMPK, and p-AMPK in the liver tissues. **(A)** FAS, Fatty acid synthase; **(B)** LPL, Lipoprotein lipase; **(C)** AMPK, Adenosine-monophosphate-activated protein kinases; **(D)** p-AMPK, phosphorylated-AMPK. The expression of β-actin was used as loading control. Relative protein expressions were quantified densitometrically with an AlphaImager 2200, and processed using AlphaEaseFC software in referring to the β-actin. HFD, high-fat diet; EM, Tween 80/Span 80 = 5:1 v/v, diluted water to 5%; AB-SCF, supercritical fluid extracted aldlay bran. C, a blank control group fed with standard chow diet and water; HFD, a high fat diet induced hyperlipidemic group fed with water; EM, a high fat diet induced fed with the emulsifier of AB-SCF; Experimental groups were induced with HFD and administrated AB-SCF/emulsifier with a dose-ascending manner, i.e., AB-SCF×0.5, 30.84 mg/kg/BW; AB-SCF×1, 61.67 mg/kg/BW; AB-SCF×5, 308.35 mg/kg/BW; AB-SCF×10, 616.70 mg/kg/BW. Data are mean ± SD, *n* = 10 hamsters in each group. Means with different letters in the same column were significantly different at *p* < 0.05 as statistically analyzed by Duncan’s multiple range tests.

To reveal the molecular-biochemical mechanism of energy metabolism exerted by AB-SCF, actions targeted on hepatic proteins from hyperlipidemic and normal hamsters, such as fatty acid synthase (FAS), lipoprotein lipase (LPL), AMPK and p-AMPK, were investigated. As results presented in the Figure 3 and Supplementary Figure S5, the expression of FAS was elevated in HFD, along with AMPK, p-AMPK were down-regulated. It is noteworthy that, just in opposite, the expression of hepatic FAS was decreased in the AB-SCF administrated hamsters with a dose-depend manner. Meanwhile, hepatic LPL, AMPK and p-AMPK had improved significantly in the both of AB-SCF×5 and AB-SCF×10 groups. These evidences suggested that the consumption of AB-SCF may exhibit the capacities in mitigating hyperlipidemia through the modulation of hepatic fat metabolism.

### Nutrition Facts in AB-SCF (per 100 g)

In Table 4, a nutrition fact list was offered. The gross energy of every 100 g AB-SCF is 793.57 ± 1.88 Kcal. Lipids (81.13 ± 0.25%) was the most abundant ingredient with no trans fats was found comprising. Proteins constituted 15.85 ± 0.15%, meanwhile, no carbohydrates, including sugar, were found in AB-SCF.

**TABLE 4:** Nutrition facts in AB-SCF (per 100 g).

AB-SCF	Ingredients
Gross energy	793.57 ± 1.88 Kcal
Lipid	81.13 ± 0.25%
Trans fats	0% (ND)
Proteins	15.85 ± 0.15%
H_2_O	2.30 ± 0.32%
Ash	0.72 ± 0.17%
Na	30.79 ± 1.07 mg
Carbohydrates, CHO	0% (N.D.)
Sugar	0% (N.D.)

AB-SCF, supercritical fluid extracted aldlay bran; N.D., not detected. The data shown are means ± SD of triplicated experiments. Analytical data was provided by the Joben Bio-Medical Co., Ltd., (Pingtung, Taiwan).

### Major Lipid Compositions and the Analytical Fingerprint of AB-SCF

**FIGURE 4:**
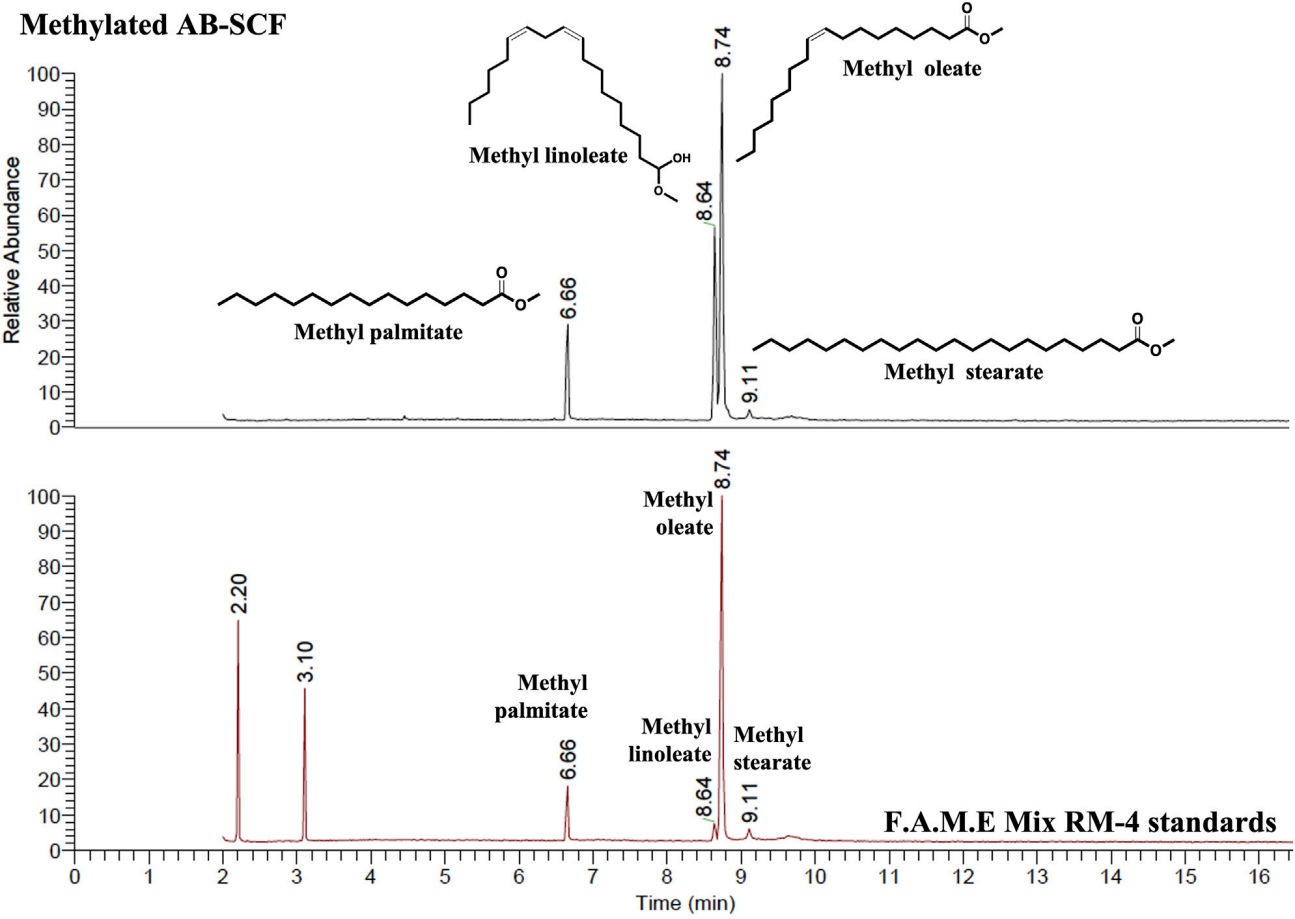
The analytical fingerprint of AB-SCF methyl esters. The analysis of Major compositions and the establishment of AB-SCF fingerprint were carried out with a gas chromatography system (Trace GC Ulture/ITQ 900, Thermo fisher Scientific, United States) with a flame ionization detector (FID). The capillary column was RT^®^-2560 (100 m × 250 μm  ×  0.2 μm) coated with biscyanopropyl polisiloxane as stationary phase (Restek Corporation, Bellefonte, PA, United States). The column oven temperature was programmed 150°C (held for 2 min), increased to 220°C at a rate of 35°C/min (held for 1 min), then raised to 225°C a rate of 0.5°C/min (maintained for 1 min). The other parameters were as follows: injection temperature, 225°C; detector temperature, 250°C; carrier gas, Helium at 1 ml/min; injection volume, 1 μl. The relative percentage of each major component in AB-SCF was quantified based on the peak area integrated by Thermo Xcalibur™ data analysis program (Thermo fisher Scientific, United States). Qualitative and quantitative analysis of AB-SCF (C16:0 Palmitate, C18:0 Stearate, C18:1 Oleate, C18:2 Linoleate) was carried out in comparing with the F.A.M.E Mix RM-4 standards.

Supercritical fluid extraction with the solvent, SC-CO_2_, was characterized by its apolar property, and known as an effective approach to obtain the oil/apolar substances. The analysis focused on the lipid compositions of AB-SCF with anti-dyslipidemic/hypercholesterolemic capacities is a considerable issue to be investigated as well. As the results presented in the Table 5, four major fatty acids, palmitic acid (C16:0; 13.07%), linoleic acid (C18:2; 28.59%), oleic acid (C18:1; 56.95%) and stearic acid (C18:0; 1.39%) were identified. Unsaturated fats majorly occupied up to 85.18% (linoleic acid + oleic acid) along with 14.16% saturated fats (palmitic acid + stearic acid) were detected. Fingerprint of AB-SCF lipids was established as shown in the Figure 4. Optimized and detailed analytic parameters for quality control using gas chromatography system were established in this work (section 2.8 and 2.9).

**TABLE 5:** Major lipid compositions of AB-SCF.

Retention time (min)	Referenced methyl esters	Ratio (%)	Identified compounds	Molecular formula	C:D*
6.66	Methyl palmitate	13.07	Palmitic acid	C_16_H_32_O_2_	C16:0
8.64	Methyl linoleate	28.59	Linoleic acid	C_18_H_32_O_2_	C18:2
8.74	Methyl oleate	56.95	Oleic acid	C_18_H_34_O_2_	C18:1
9.11	Methyl stearate	1.39	Stearic acid	C_18_H_36_O_2_	C18:0

The analysis of Major compositions and the establishment of AB-SCF fingerprint were carried out with a gas chromatography system (Trace GC Ulture/ITQ 900, Thermo fisher Scientific, United States) with a flame ionization detector (FID). The capillary column was RT®-2560 (100 m × 250 μm × 0.2 μm) coated with biscyanopropyl polisiloxane as stationary phase (Restek Corporation, Bellefonte, PA, United States). The column oven temperature was programmed 150°C (held for 2 min), increased to 220°C at a rate of 35°C/min (held for 1 min), then raised to 225°C a rate of 0.5°C/min (maintained for 1 min). The other parameters were as follows: injection temperature, 225°C; detector temperature, 250°C; carrier gas, Helium at 1 ml/min; injection volume, 1 μl. The relative percentage of each major component in AB-SCF was quantified based on the peak area integrated by Thermo Xcalibur™ data analysis program (Thermo fisher Scientific, United States). Qualitative and quantitative analysis of AB-SCF (C16:0 Palmitate, C18:0 Stearate, C18:1 Oleate, C18:2 Linoleate) was carried out in comparing with the F.A.M.E Mix RM-4 standards. *C:D is the numerical symbol: total amount of (C)arbon atoms of the fatty acid, and the number of (D)ouble (unsaturated) bonds in it; if D > 1 it is assumed that the double bonds are separated by one or more methylene bridge(s).

### General Separation Processes and Identification of the Isolated Compounds From AB-SCF

**FIGURE 5:**
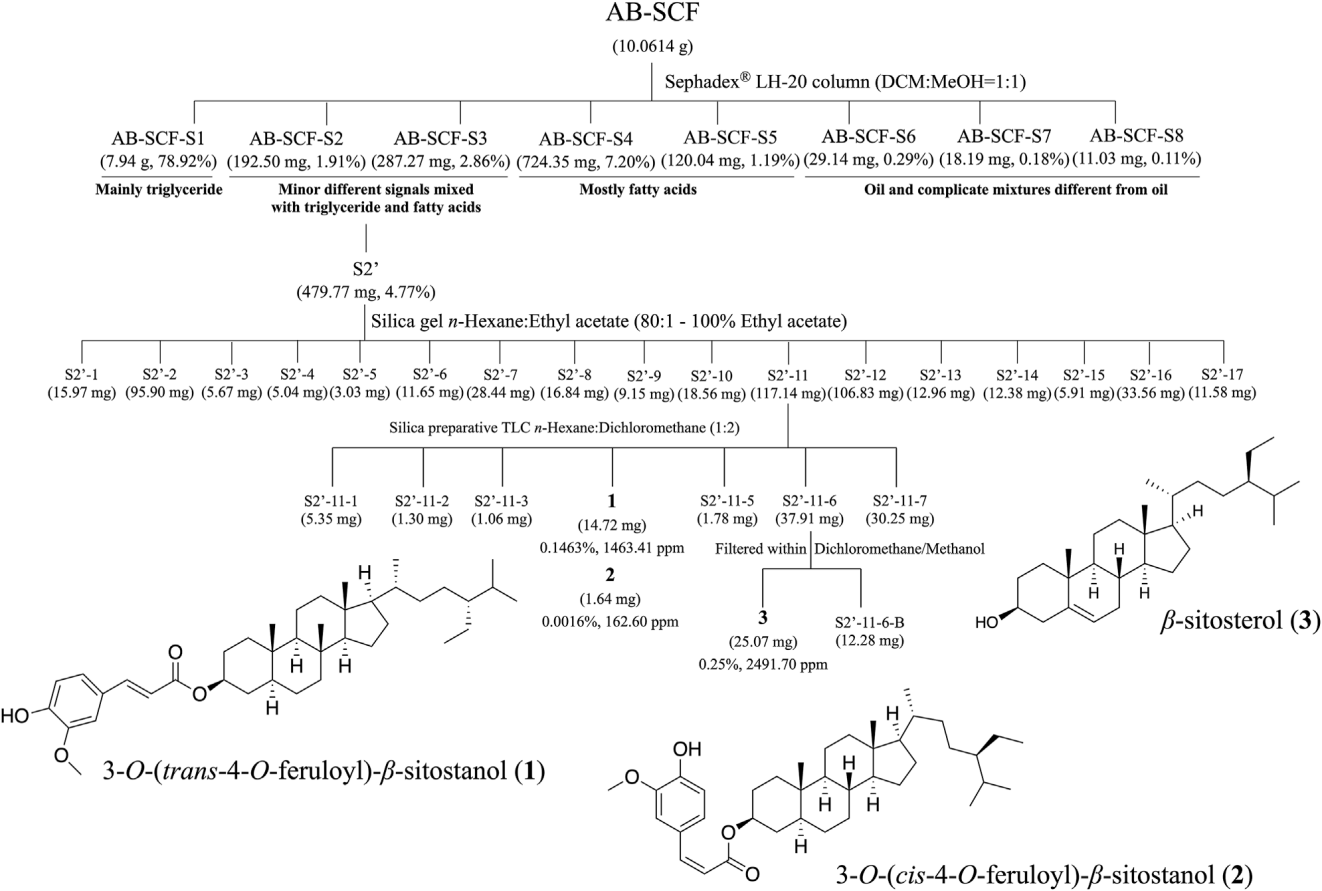
The separation processes and isolated compounds of non-lipid constituents from AB-SCF. AB-SCF was first size-meshed through Sephadex^®^ LH-20 to be pre-divided from fatty acids. AB-SCF-S2 and S3 were detected with minor signals different from lipid in ^1^H NMR screens, and combined as S2′ for further SiO_2_ column chromatography and preparative TLC separations and purifications. Three non-lipid pure compounds, 3-*O*-(*trans*-4-feruloyl)-*β*-sitostanol (**1**), 3-*O*-(*cis*-4-feruloyl)-*β*-sitostanol (**2**) and *β*-sitosterol (**3**), were obtained, and identified based on MS, ^1^H NMR, ^13^C NMR and comparison with authentic samples or with published data.

The complete flow chart for separation and isolated pure compounds from AB-SCF were presented in the Figure 5. Detailed chromatographic works were recorded in Supplementary Figures S1–S4 as described in the section 2.9. Three compounds, 3-*O*-(*trans*-4-feruloyl)-*β*-sitostanol (**1**) (Condo et al., 2001), 3-*O*-(*cis*-4-feruloyl)-*β*-sitostanol (**2**) (Akihisa et al., 2000) and *β*-sitosterol (**3**) (Chaturvedula and Prakash., 2012), were the obtained with the yields of 14.724 mg (0.1463%, 1463.41 ppm), 1.636 mg (0.0016%, 162.60 ppm) and 25.07 mg (0.2491%, 2491.70 ppm), respectively. Each compound was identified by MS, ^1^H NMR, ^13^C NMR and comparison with authentic samples or with published data.

3-*O*-(trans-4 feruloyl)-*β*-sitostanol (**1**): EISMS *m/z* 591.71 [M-H]^−^. ^1^H NMR (CDCl_3_): *δ*_H_ 0.6–2.0 (*β*-sitostanol moiety, ∼50H), 3.91 (s, 3H), 4.82 (tt, *J* = 11.0, 4.8 Hz, 1H), 5.88 (s, 1H), 6.27 (d, *J* = 15.9 Hz, 1H), 6.91 (d, *J* = 8.1 Hz, 1H), 7.03 (d, J = 1.7, 1H), 7.06 (dd, 8.2, 1.8, 1H), 7.59 (d, *J* = 15.9 Hz, 1H). ^13^C NMR: 11.96, 12.05, 12.24, 18.71, 19.01, 19.80, 21.29, 23.04, 24.20, 26.05, 27.62, 28.25, 28.62, 29.12, 31.99, 33.90, 34.16, 35.48, 35.48, 36.16, 36.78, 39.96, 42.57, 44.67, 45.81, 54.22, 55.89, 56.15, 56.40, 73.71, 109.22, 114.66, 116.18, 122.98, 127.11, 144.36, 146.71, 147.79, 166.81 (Supplementary Figure S6).

The ^1^H NMR (CDCl_3_) data of 3-*O*-(*cis*-4-feruloyl)-*β*-sitostanol (**2**), a rotated geometric isomer minorly mixed in (**1**) were listed as follows (Supplementary Figure S7): δ 7.11 (d, *J* = 8.2 Hz, 3H), 6.87 (d, *J* = 8.2 Hz, 2H), 6.76 (d, *J* = 13.0 Hz, 2H), 6.47 (d, *J* = 15.9 Hz, 1H), 6.06 (d, *J* = 15.9 Hz, 0H), 5.80 (d, *J* = 4.0 Hz, 1H), 5.77 (d, *J* = 4.2 Hz, 1H), 5.39 (s, 1H), 4.37 (q, *J* = 7.2 Hz, 1H), 3.65 (s, 3H). (**1**) and (**2**) would interconvert to be each other, and steadily exist with a ratio of 9:1 observed according to the integral quantities of ^1^H signals.

*β*-sitosterol (**3**): EIMS m/z 414.72 [M^+^]. ^1^H NMR (CDCl_3_): H-3 (*δ*_H_ 3.55, dtt, *J =* 27.0, 10.9, 4.6 Hz, 1H), H-5 (*δ*_H_ 5.34, m, 1H), H-19 (*δ*_H_ 0.91, d, *J =* 6.6 Hz, 3H), H-24 (*δ*_H_ 0.85, d, *J =* 7.6 Hz, 3H), H-26 (*δ*_H_ 0.82, s, 3H), H-27 (*δ*_H_ 0.80, s, 3H), H-28 (*δ*_H_ 0.67, s, 3H), H-29 (*δ*_H_ 1.00, s, 3H). ^13^C NMR: 11.99, 12.21, 18.91, 19.16, 19.53, 19.95, 21.21, 23.19, 24.43, 28.38, 29.28, 31.78, 32.04, 36.64, 37.39, 39.91, 40.63, 42.42, 42.45, 45.96, 50.26, 56.19, 56.90, 71.92, 77.00, 121.84, 129.41, 138.46, 140.91 (Supplementary Figure S8).

^1^H NMR and ^13^C NMR spectra were performed on JEOL JNM-ECS 400 MHz NMR Spectrometer (^1^H, F400 MHz; ^13^C, 100 MHz) and Varian Mercury Plus 400 MHz FT-NMR (^1^H, 400 MHz; ^13^C, 100 MHz) in CDCl_3_. Mass spectra were obtained from Waters 2695 Separations Module (ESI-MS).

## Discussion

In this work, high-fat diet severely induced hyperlipidemia-related syndromes on hamsters, i.e., higher BW, serum TG, TC, LDL-C, lowered HDL-C; and hepatic lipid accumulation (TG and TC) in hamsters, which situations were consistent with similar models of previous investigations (Rideout et al., 2014; Wang et al., 2011). EM, an HFD group administrated with the emulsifier prepared as vehicle (Tween 80/Span 80 in the ratio of 5:1 v/v, then diluted with sterilized RO water to 5%) for AB-SCF did not influent the induction of HFD on hamsters.

After 8 weeks of administration of AB-SCF×0.5, AB-SCF×1, AB-SCF×5 and AB-SCF×10 to hyperglycemic hamsters, AB-SCF×10 displayed a significant prevention of dramatic body weight gains, and stably remain it close to the normal group. Higher dose of AB-SCF may exert anti-obesity properties. All AB-SCF groups exhibited markedly lower data than HFD-induced group in not only serum TG and TC, but also in LDL-C. Since HDL-C levels were ascendingly improved, LDL-C/HDL-C ratios, the predictor of cardiovascular risk (Jukema et al., 2005), were meaningfully ameliorated by AB-SCF as well. In feces, no obvious difference in lipid and lipoprotein profiles were detected. However, AB-SCF groups would improve the excretions of bile acids with significances. These positive responses of serum biochemical data and fecal bile acids were all occurred in a dose-dependent manner. To the assessments of hepatic TG, TC, AB-SCF had significantly ameliorated the levels of hepatic TG and TC. Especially in the AB-SCF×10 group, these two indicators were almost retrieved back to the levels of the standard chow diet hamsters. AB-SCF may also be considered as an alleviating agent against lipid peroxidation in liver due to the proper regulation of tissue MDA and GSH.

According to the results of chemical constituent analysis in this work, lipid related components in AB-SCF, e.g., triglyceride, fatty acids (saturated fat and unsaturated fat) were occupied at least 80% in the investigations of nutrition facts (Table 4) and the separation processes (Figure 5). Although there was no direct report focused on the ingredients of AB-SCF, adlay was once indicated containing a significantly abundant amount of lipids than most of the common cereals (Xi et al., 2016). Unsaturated fatty acids, composed by linoleic acid and oleic acid, were totally detected up to 85.54% in the major lipid compositions of AB-SCF. Previous studies have revealed that linoleic acid would reduce the level of LDL-C and enhance the level of HDL-C in hamsters (Valeille et al., 2004; Lock et al., 2005). Furthermore, linoleic acid was descripted to exhibit health effects against obesity and other diseases of lipid metabolism (Shen and McIntosh, 2016). Linoleic acid which exists 28.59% in the AB-SCF may be suggested as being responsible for the beneficial effects of lipid metabolism disorders carried out in this study. Oleic acid, 56.95% comprised in the AB-SCF, would improve lipid profile. The supplementation with oleic acid showed a beneficial effect on antioxidant capacity related to components of metabolic syndrome (Pastor et al., 2021). It was also proved against hepatic ischemia and reperfusion injury in mice and would able to reduce the amount of intracellular ROS due to the enhancement of intracellular GSH production and the limit of intracellular lipid peroxidation levels induced by H_2_O_2_ (Guo et al., 2019). Meanwhile, linoleic acid may also enhance GSH content through an induction of gamma-glutamylcysteine ligase (Arab et al., 2006). Linoleic acid and oleic acid can be considered as the key for alleviating lipid peroxidation in liver due to the positive responses in tissue MDA and GSH assessments.

3-*O*-(*trans*-4-feruloyl)-*β*-sitostanol (**1**) and 3-*O*-(*cis*-4-feruloyl)-*β*-sitostanol (**2**) were isolated compounds from non-lipid partitioned fraction of AB-SCF are ferulate phytostanol esters (phytostanols). They were known to lower LDL-C levels in humans by up to 15% by inhibiting the absorption of cholesterol from the intestine. USFDA has permitted a rare health claim for their use in low-fat diets (Condo et al., 2001). On the other hand, the structure of *β*-sitostanol (**3**) was similar to that of cholesterol. It was even long known about its hypocholesterolemia capacity (Zák et al., 1990). Phytosterol and phytostanols were reported to increase bile acid excretion (Becker et al., 1993; Normén et al., 2000). Since bile acid was a metabolite from cholesterol, it would be secreted to intestine from liver to assist in the digestion of intake fat. The increase of bile acid expend would lead to more cholesterol metabolized in liver for filling up the spent bile acid. 3-*O*-(*trans*-4-feruloyl)-*β*-sitostanol, 3-*O*-(*cis*-4-feruloyl)-*β*-sitostanol and *β*-sitostanol summed as 0.41% (4117.72 ppm), along with linoleic acid and oleic acid (85.54%) in the AB-SCF may play an important role in the enhancement of cholesterol metabolism and bile acid excretion. These compositions may synergistically trigger the mentioned anti-dyslipidemic/hypercholesterolemic capacities of AB-SCF.

To the bio-molecular mechanism of hepatic energy metabolism, AB-SCF which exhibited the capacities in activating AMPK and p-AMPK may not only suppress the syntheses of fatty acids and cholesterol, but also reduce the hepatic gluconeogenesis and insulin resistance (Viollet et al., 2009; Liu et al., 2017). Through AMPK pathway, AB-SCF would also regulate lipid oxidation and hepatic lipid accumulation in liver (Long and Zierath, 2006). AB-SCF which also increase the hepatic LPL would impact on the hydrolysis of circulating TG, chylomicrons and VLDL (very low-density lipoprotein). AB-SCF may play a role in lipoprotein metabolism activated to launch hypolipidemic effect and prevention of atherogenesis (Santamarina-Fojo et al., 2004; Wang and Eckel, 2009). FAS which down-regulated by AB-SCF is a key enzyme in lipogenesis. The suppression of hepatic FAS would inhibit the fatty acid and TG synthesis in HFD-induced hamster owing to the catalysis of acetyl-CoA and malonyl-CoA (Engin, 2017; Rideout et al., 2014).

## Conclusion

In the current study, AB-SCF exhibited a lipid-regulating potential on hyperlipidemic hamsters by preventing the body weight gain, ameliorating the elevation of serum TG, TC and LDL-C levels, as well as improving the rises of hepatic TG and TC levels. HDL-C was enhanced, along with the attenuation on the crucial predictor of cardiovascular risk, the LDL-C/HDL-C ratios. The energy metabolic mechanisms were clarified with the down-regulation of FAS, along with the up-regulations of LPL, AMPK and p-AMPK proteins in liver tissues. These results were exhibited with a dose-dependent manner. The active ingredients of AB-SCF were indicated and composed by linoleic acid, oleic acid, 3-*O*-(*trans*-4-feruloyl)-*β*-sitostanol, 3-*O*-(*cis*-4-feruloyl)-*β*-sitostanol and *β*-sitostanol. These compositions may be synergistically responsible for the anti-dyslipidemic/hypercholesterolemic capacities of AB-SCF. They can also be monitored as crucial standards in quality control.

The evidences carried out in this work brought out a hint for further utilization aiming at the byproduct generated during the refining processes of polished adlay. AB-SCF may be considered as a promising complementary supplement, and developed as a functional food or new botanical drug in the future.

## Data Availability

The raw data supporting the conclusions of this article will be made available by the authors, without undue reservation.
