# Supplementary material for: Lipid Metabolism and its Mechanism Triggered by Supercritical CO2 Extract of Adlay (Coix lacryma-jobi var. ma-yuen (Rom. Caill.) Stapf) Bran in High-Fat Diet Induced Hyperlipidemic Hamsters
Source: Front Pharmacol. 2021 Nov 17;12:785944. doi: 10.3389/fphar.2021.785944 (PMC8635772; doi:10.3389/fphar.2021.785944)

Supplementary Material

Lipid metabolism and its mechanism triggered by supercritical CO_2 extract of adlay (*Coix lacryma-jobi var. ma-yuen* (Rom. Caill.) Stapf) bran in high fat diet-induced hyperlipidemic hamsters

Chiao-Chih Huang[1], Tzu-Ching Lin[1], Chiung-Hui Liu[1], Hao-Chun Hu[1], Szu-Yin Yu[1], Shu-Jing Wu[2], Ming-Hong Yen[1], Yi-Hong Tsai[1,3*], Fang-Rong Chang[1,4,5,6,*]

[1] Graduate Institute of Natural Products, College of Pharmacy, Kaohsiung Medical University, Kaohsiung 80708, Taiwan
[2] Department of Nutritional Health, Chia-Nan University of Pharmacy and Science, Tainan 71710, Taiwan
[3] Department of Pharmacy and Master Program, Collage of Pharmacy and Health Care, Tajen University, Pingtung County 90741, Taiwan.
[4] Drug Development and Value Creation Research Center, Kaohsiung Medical University, Kaohsiung 80708, Taiwan
[5] Department of Medical Research, Kaohsiung Medical University Hospital, Kaohsiung Medical University, Kaohsiung 80708, Taiwan
[6] Department of Marine Biotechnology and Resources, National Sun Yat-sen University, Kaohsiung 804, Taiwan

*** Correspondence:**
Fang-Rong Chang
aaronfrc@kmu.edu.tw

Yi-Hong Tsai
lyph0719@hotmail.com

Contents

Fig S1. TLC observation of AB-SCF-S1 to S8 .... **3**

Fig S2. Brief screen of ^{1}H NMR spectra on every AB-SCF-Sn fraction **4**

Fig S3. Detail separation scheme of **1**, **2** and **3** **5**

Fig S4. Preparative TLC operation of S2'-11 .... **6**

Fig S5. Triplicated western blot analysis of liver tissue proteins **7**

Fig S6. ^{1}H NMR and ^{13}C NMR of 3-*O*-(*trans*-4-feruloyl)-*β*-sitostanol (**1**) **8**

Fig S7. ^{1}H NMR and ^{13}C NMR of 3-*O*-(*cis*-4-feruloyl)-*β*-sitostanol (**2**) **9**

Fig S8. ^{1}H NMR and ^{13}C NMR of *β*-sitosterol (**3**) **10**

Figure S1. TLC observation of AB-SCF-S1 to S8.

AB-SCF
(10.0614 g)

Sephadex® LH-20 column (DCM:MeOH=1:1)

AB-SCF-S1	AB-SCF-S2	AB-SCF-S3	AB-SCF-S4	AB-SCF-S5	AB-SCF-S6	AB-SCF-S7	AB-SCF-S8
(7.94 g, 78.92%)	(192.50 mg, 1.91%)	(287.27 mg, 2.86%)	(724.35 mg, 7.20%)	(120.04 mg, 1.19%)	(29.14 mg, 0.29%)	(18.19 mg, 0.18%)	(11.03 mg, 0.11%)

TLC mobile phase = *n*-Hexane : Ethyl acetate = 5:1

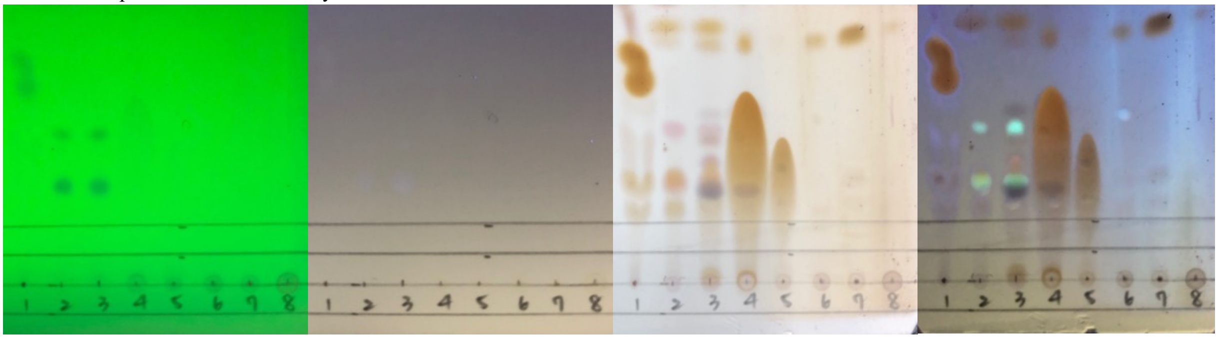

UV = 254 nm   UV = 360 nm   UV = 254 nm   UV = 360 nm

visualized by 10% (v/v) H_2SO_4 / ethanol spray

Figure S2. Brief screen of ^{1}H NMR spectra on every AB-SCF-Sn fractions.

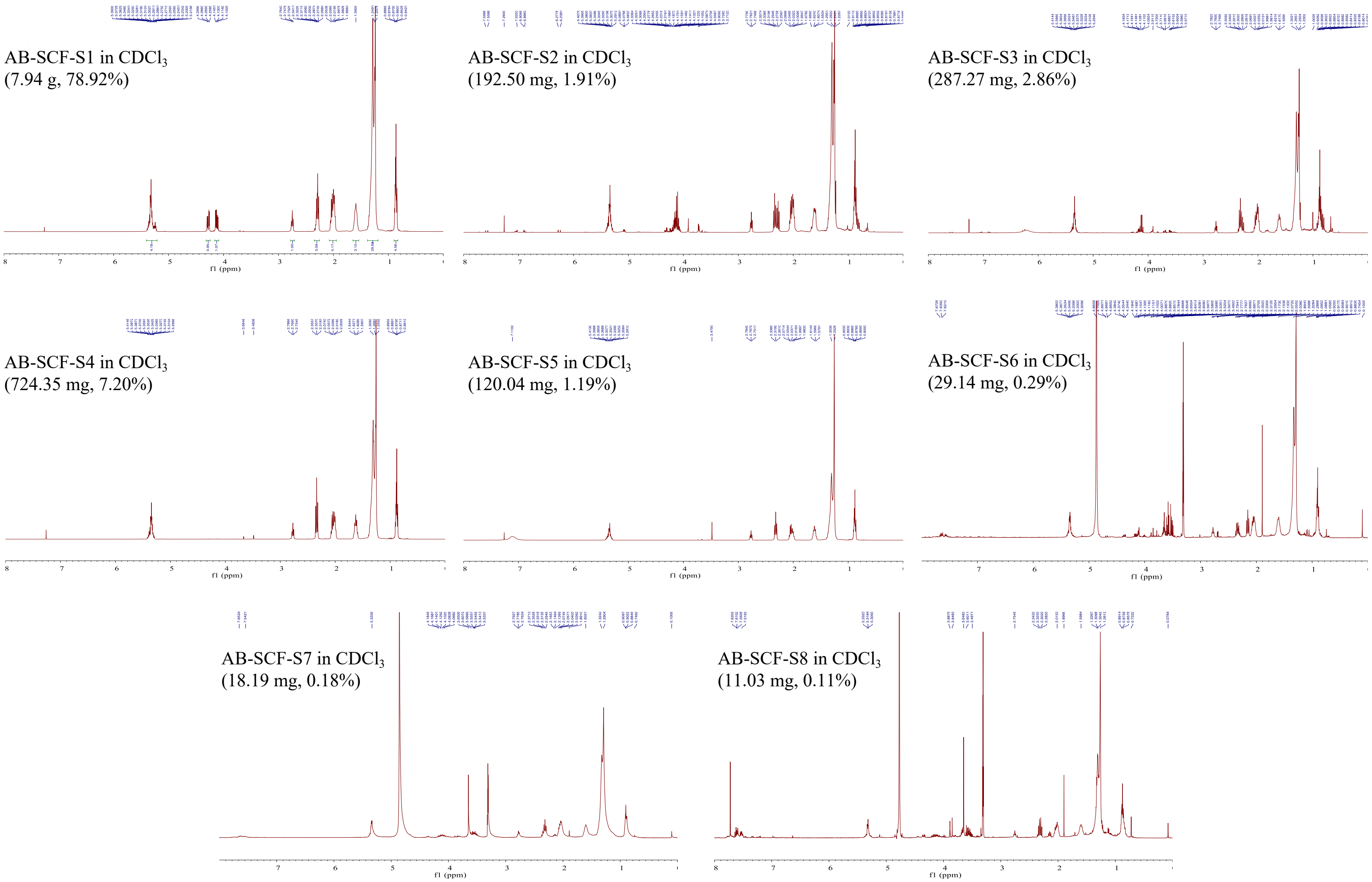

Figure S3. Detail separation scheme of **1**, **2** and **3**.

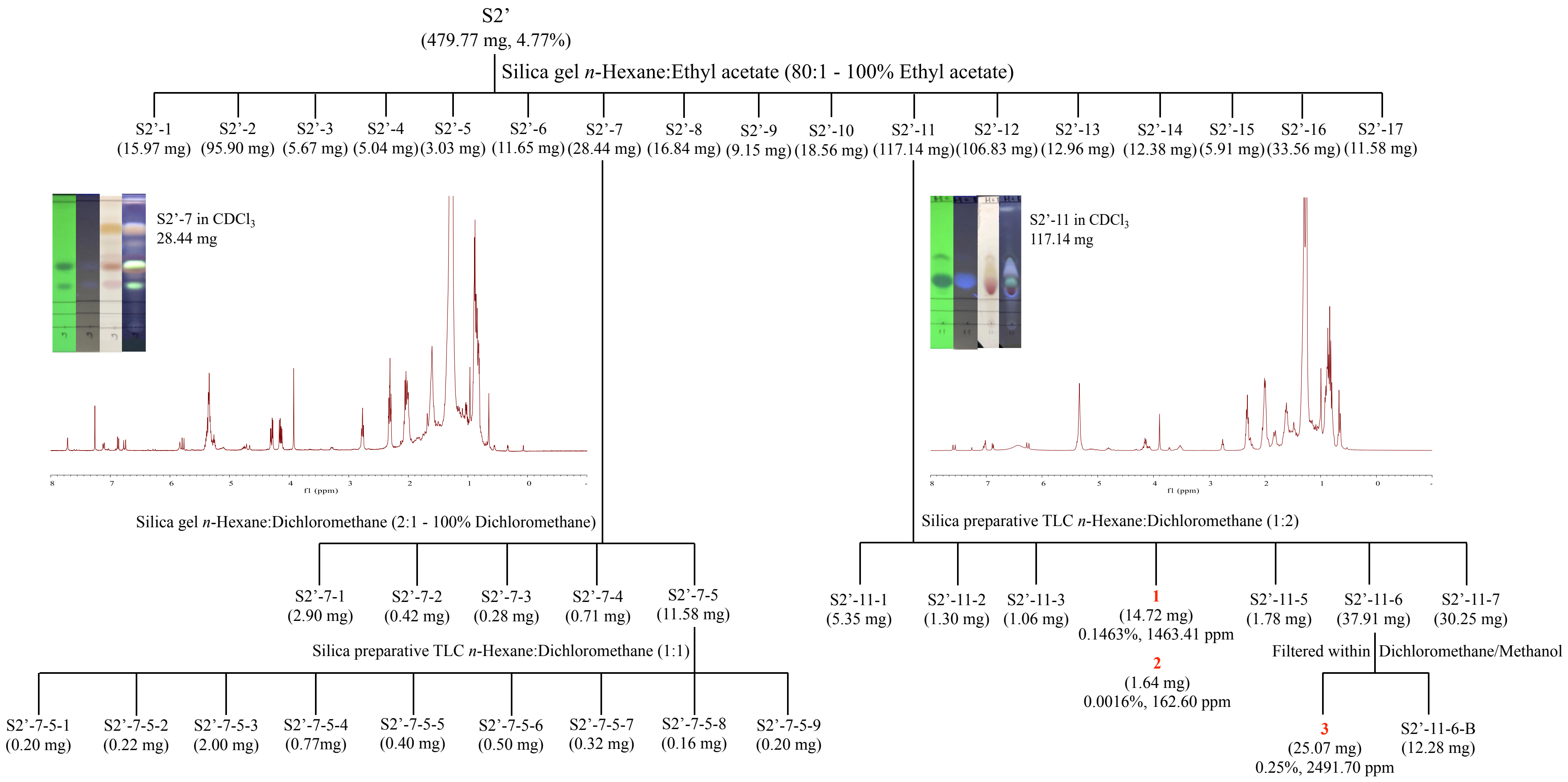

Figure S4. Preparative TLC operation of S2'-11.

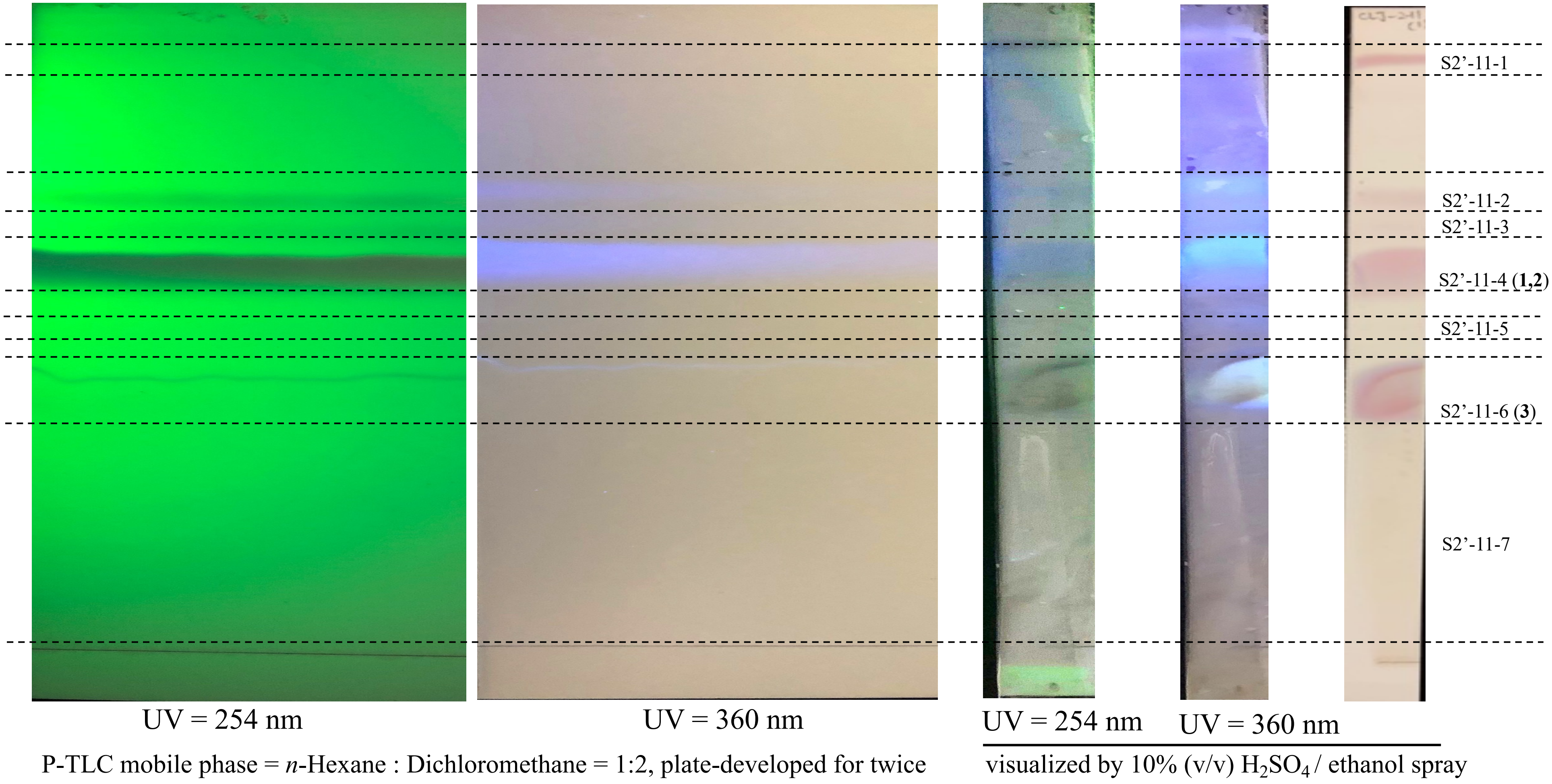

Figure S5. Triplicated western blot analysis of liver tissue proteins.

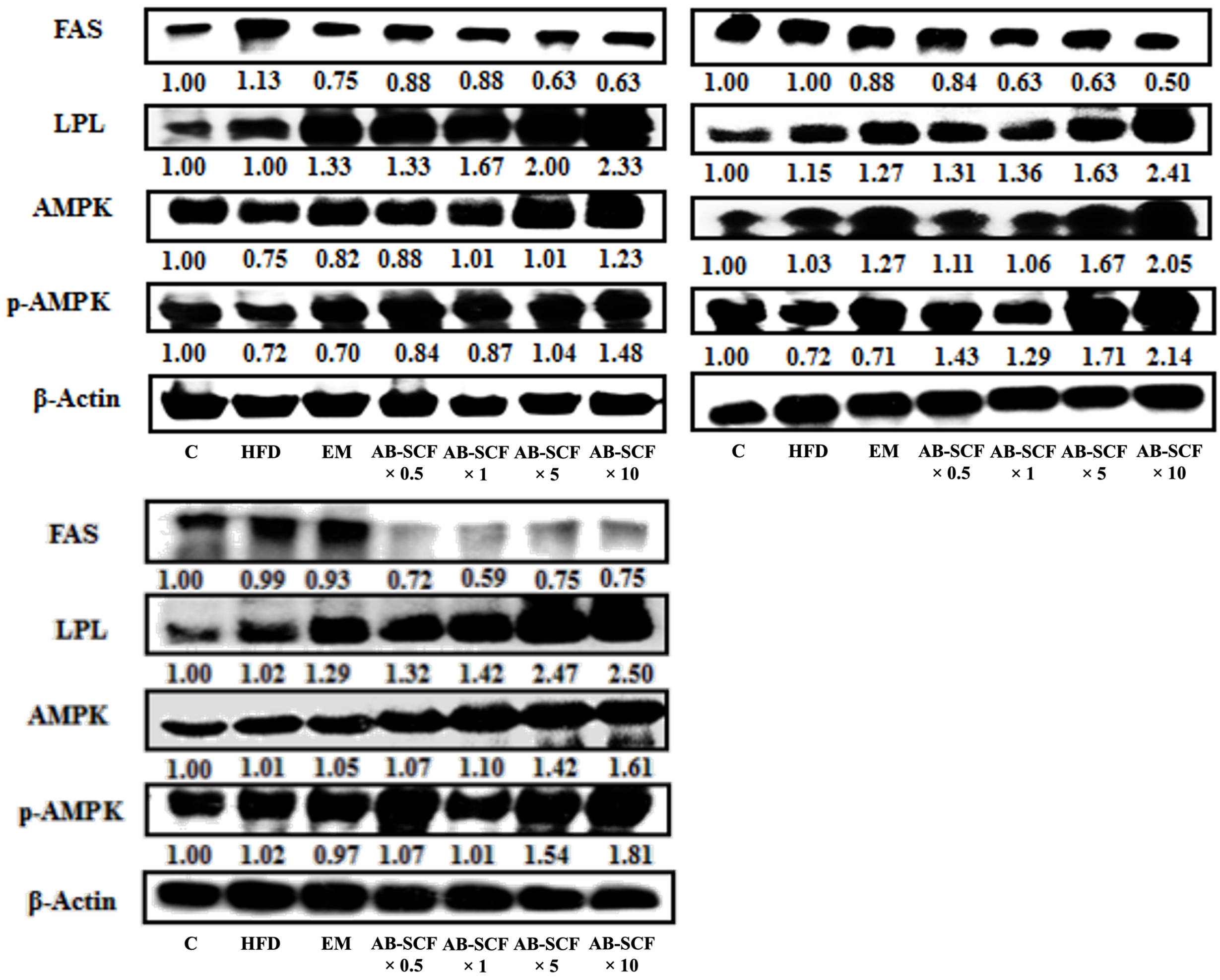

Figure S6. ^{1}H NMR and ^{13}C NMR of of 3-*O*-(*trans*-4-feruloyl)-*β*-sitostanol (**1**).

^{1}**H-NMR**

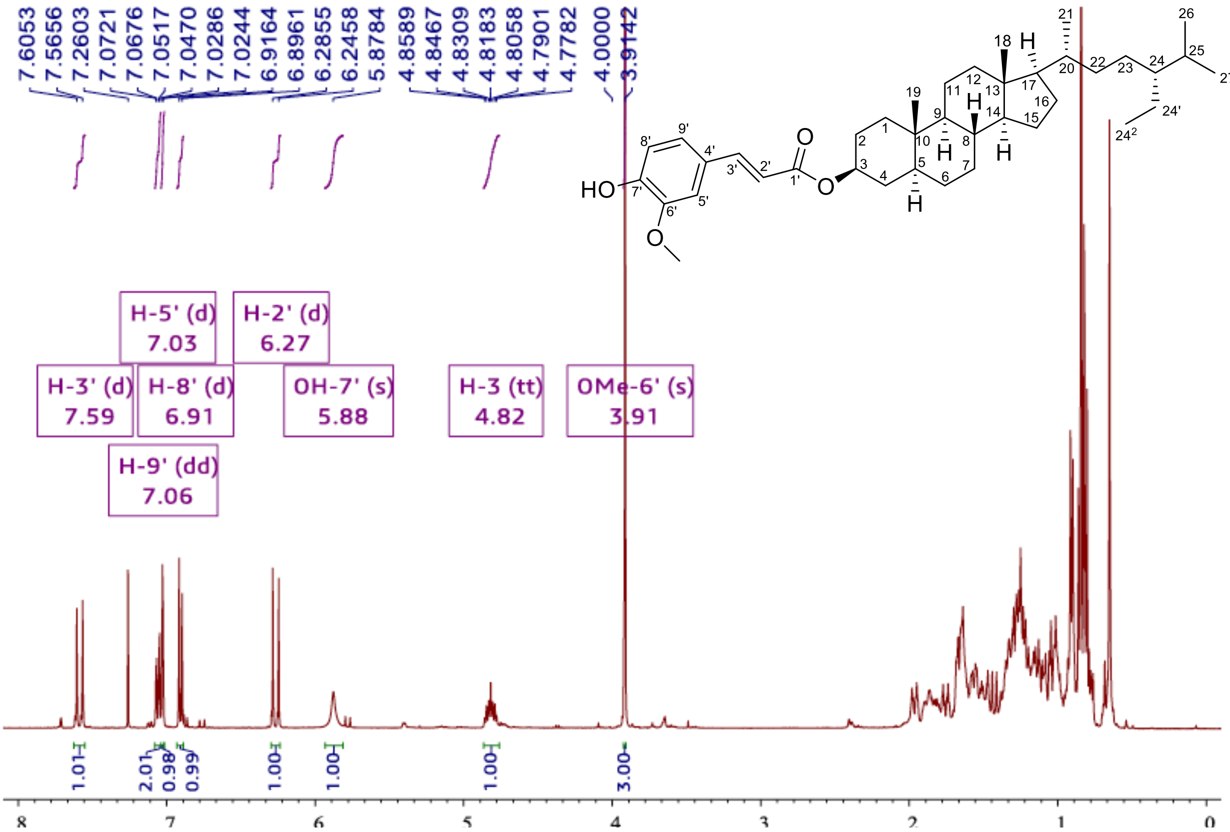

^{13}C NMR

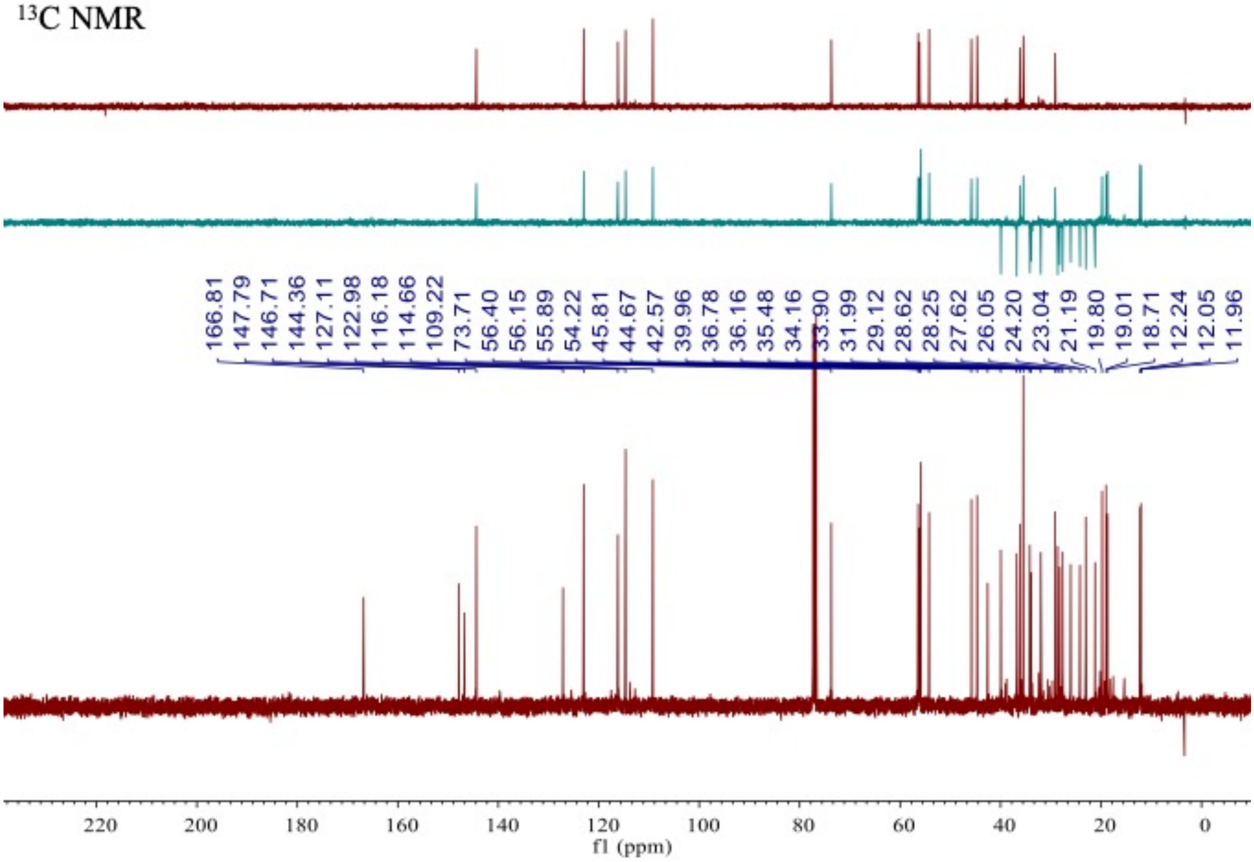

Figure S7. ^{1}H NMR and ^{13}C NMR of of 3-*O*-(*cis*-4-feruloyl)-*β*-sitostanol (**2**).

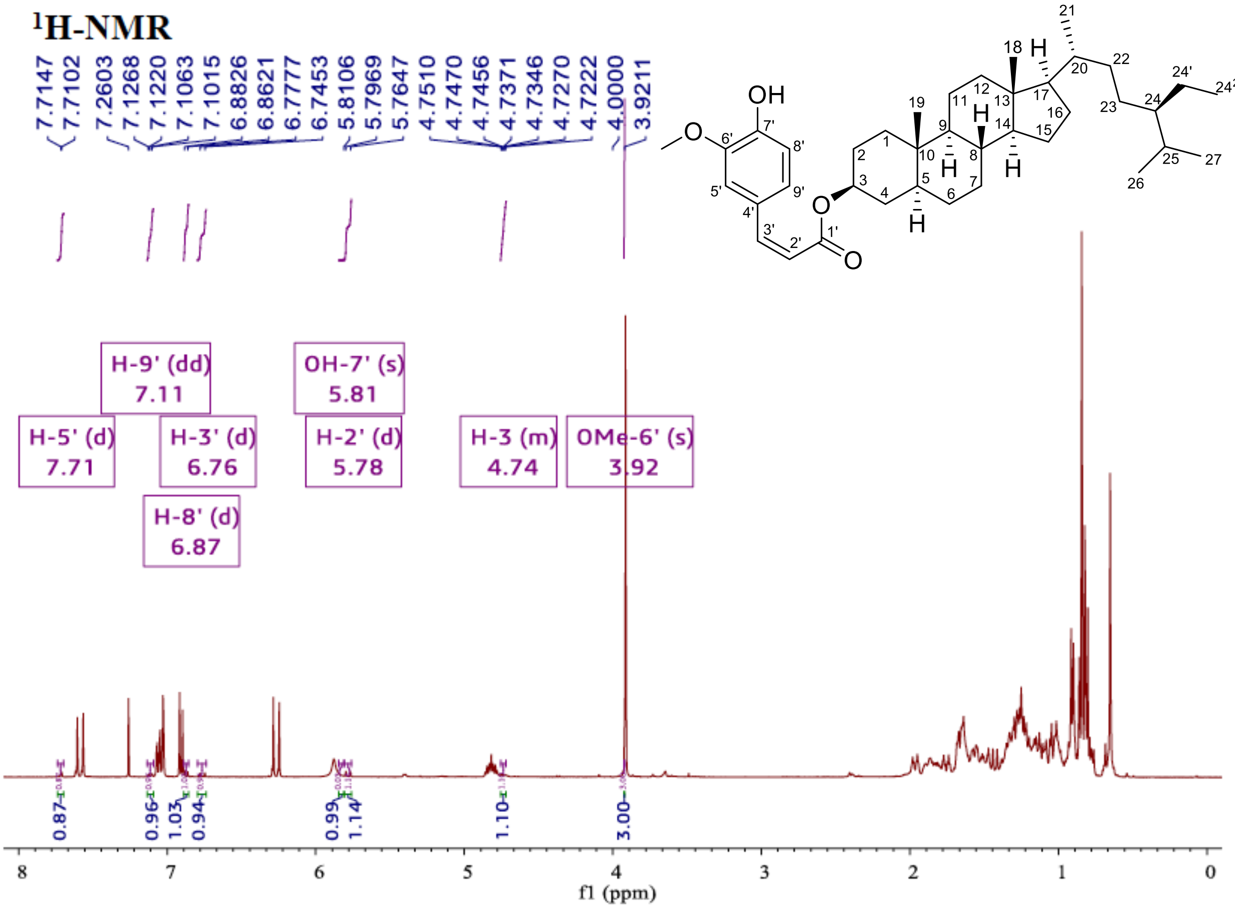

Figure S8. ^{1}H NMR and ^{13}C NMR of *β*-sitosterol (**3**).

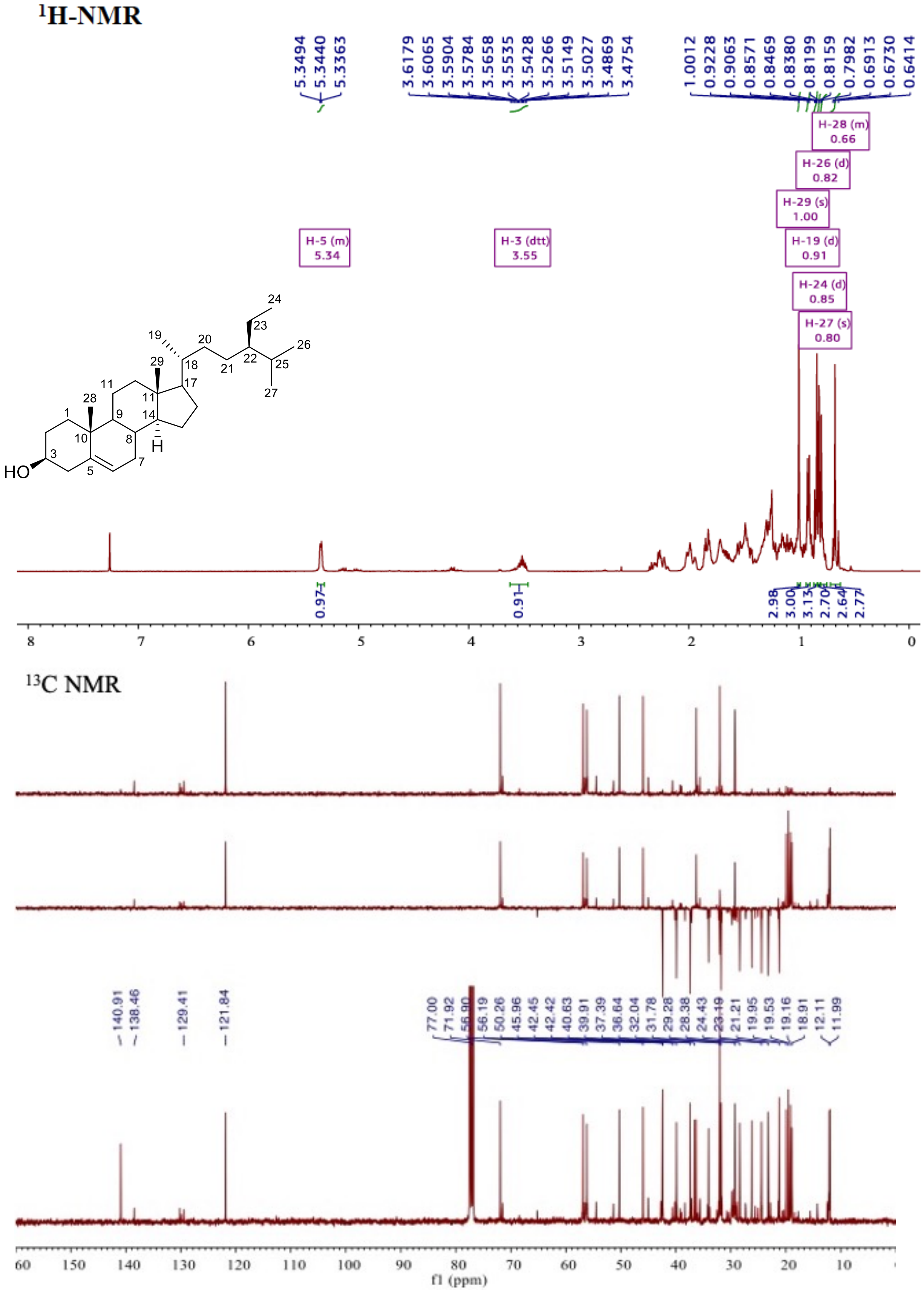

Supplement: Supplementary file 1 [file DataSheet1.PDF]
